# A platinum standard pan-genome resource that represents the population structure of Asian rice

**DOI:** 10.1038/s41597-020-0438-2

**Published:** 2020-04-07

**Authors:** Yong Zhou, Dmytro Chebotarov, Dave Kudrna, Victor Llaca, Seunghee Lee, Shanmugam Rajasekar, Nahed Mohammed, Noor Al-Bader, Chandler Sobel-Sorenson, Praveena Parakkal, Lady Johanna Arbelaez, Natalia Franco, Nickolai Alexandrov, N. Ruaraidh Sackville Hamilton, Hei Leung, Ramil Mauleon, Mathias Lorieux, Andrea Zuccolo, Kenneth McNally, Jianwei Zhang, Rod A. Wing

**Affiliations:** 1 Center for Desert Agriculture, Biological and Environmental Sciences & Engineering Division (BESE), King Abdullah University of Science and Technology (KAUST), Thuwal, 23955-6900 Saudi Arabia; 2 International Rice Research Institute (IRRI), Strategic Innovation, Los Baños, 4031 Laguna Philippines; 3 Arizona Genomics Institute, School of Plant Sciences, University of Arizona, Tucson, Arizona 85721 USA; 4 Genomics Technologies, Applied Science and Technology, Corteva AgriscienceTM, Iowa, IA 50131 USA; 5 Rice Genetics and Genomics Lab, International Center for Tropical Agriculture (CIAT), Cali, Colombia; 6 University of Montpellier, DIADE, IRD, Montpellier, France; 7 Institute of Life Sciences, Scuola Superiore Sant’Anna, Pisa, Italy; 8 National Key Laboratory of Crop Genetic Improvement, Huazhong Agricultural University, Wuhan, 430070 China

**Keywords:** Agriculture, Plant genetics, DNA sequencing, Structural variation, Evolutionary genetics

## Abstract

As the human population grows from 7.8 billion to 10 billion over the next 30 years, breeders must do everything possible to create crops that are highly productive and nutritious, while simultaneously having less of an environmental footprint. Rice will play a critical role in meeting this demand and thus, knowledge of the full repertoire of genetic diversity that exists in germplasm banks across the globe is required. To meet this demand, we describe the generation, validation and preliminary analyses of transposable element and long-range structural variation content of 12 near-gap-free reference genome sequences (RefSeqs) from representatives of 12 of 15 subpopulations of cultivated Asian rice. When combined with 4 existing RefSeqs, that represent the 3 remaining rice subpopulations and the largest admixed population, this collection of 16 Platinum Standard RefSeqs (PSRefSeq) can be used as a template to map resequencing data to detect virtually all standing natural variation that exists in the pan-genome of cultivated Asian rice.

## Abstract (summary)

**Table:** 

Measurement(s)	genome • DNA • sequence_assembly • sequence feature annotation • physical map
Technology Type(s)	DNA sequencing • PacBio Sequel System • sequence assembly process • transposable elements annotation • Optical Mapping Illumina sequencing
Factor Type(s)	Oryza sativa cv. variety
Sample Characteristic - Organism	Oryza sativa

Machine-accessible metadata file describing the reported data: 10.6084/m9.figshare.11950596

## Background & Summary

Asian cultivated rice is a staple food for half of the world population. With the planet’s population expected to reach 10 billion by 2050, farmers must increase production by at least 100 million metric tons per year^1,2^. To address this need, future rice cultivars should provide higher yields, be more nutritious, be resilient to multiple abiotic and biotic stresses, and have less of an environmental footprint^3,4^. To achieve this goal, a comprehensive and more in-depth understanding of the full range of genetic diversity of the pan-cultivated rice genome and its wild relatives will be needed^5^.

With a genome size of ~390 Mb, rice has the smallest genome among the domesticated cereals, making it particularly amenable to genomic studies^6^ and the primary reason why it was the first crop genome to be sequenced 15 years ago^6,7^. To better understand the full-range of genetic diversity that is stored in rice germplasm banks around the world, several studies have been conducted using microarrays^8,9^ and low coverage skim sequencing^10,11^. In 2018, a detailed analysis of the Illumina resequencing of more than 3,000 diverse rice accessions (a.k.a. 3K-RG), aligned to the *O. sativa* v.g. japonica cv. Nipponbare reference genome sequence (a.k.a. IRGSP RefSeq), showed how the high genetic diversity present in domesticated rice populations provides a solid base for the improvement of rice cultivars^12^. One key finding from a population structure analysis of this dataset showed that the 3,000 accessions can be subdivided into nine subpopulations, where most accessions from close sub-groups could be associated to geographic origin^12^.

One critical piece of information missing from these analyses is the fact that single nucleotide polymorphisms (SNPs) and structural variations (SVs) present in subpopulation specific genomic regions have yet to be detected because the 3K-RG data set was only aligned to a single reference genome. Therefore, the next logical step, to capture and understand genetic variation pan-subpopulation-wide is to map the 3K-RG dataset to high-quality reference genomes that represent each of the subpopulations of cultivated Asian rice. At present, only a handful high-quality rice genomes for cultivated rice are publicly available^5,6,13,14^, thus, there is an immediate need for such a comprehensive resource to be created, which is the subject of this Data Descriptor.

Here we present a reanalysis of the population structure analysis discussed above^12^ and show that the 3K-RG dataset can be further subdivided into a total of 15 subpopulations. We then present the generation of 12 new and near-gap-free high-quality PacBio long-read reference genomes from representative accessions of the 12 subpopulations of cultivated Asian rice for which no high-quality reference genomes exist. All 12 genomes were assembled with more than 100x genome coverage PacBio long-read sequence data and then validated with Bionano optical maps^15^. The number of contigs covering each of the twelve assemblies, excluding unplaced contigs, ranged from 15 (GOBOL SAIL (BALAM)::IRGC 26624-2) to 104 (IR 64). The contig N50 value for the 12-genome dataset ranged from 7.35 Mb to 31.91 Mb. When combined with 4 previously published genomes (*i.e*. Minghui 63 (MH 63), Zhenshan 97 (ZS 97)^13,14^, N 22^5^ and the IRGSP RefSeq.^6^), this 16-genome dataset can be used to represent the K = 15 population/admixture structure of cultivated Asian rice.

## Methods

### Ethics statement

This work was approved by the University of Arizona (UA), the King Abdullah University of Science and Technology (KAUST), Huazhong Agricultural University (HZAU), the International Rice Research Institute (IRRI) and the International Center for Tropical Agriculture (CIAT). All methods used in this study were carried out following approved guidelines.

### Population structure

We extracted 30 subsets of 100,000 randomly chosen SNPs out of the 3K-RG Core SNP set v0.4 (996,009 SNPs, available at https://snp-seek.irri.org/_download.zul). For each subset, we ran ADMIXTURE^16^ with the number of ancestral groups K ranging from 5 to 15. We then aligned the resulting Q matrices using CLUMPP software^17^. Since different runs at a given value of K often give rise to different refinements (splits) of the lower level grouping, we first clustered the runs for each K according to similarity of Q matrices using hierarchical clustering, thus obtaining several clusters of runs for each K. We discarded one-element clusters (outlier runs), and averaged the Q matrices within each remaining cluster. Figure S1 shows the admixture proportions taken from the averaged Q matrices of the final clusters for K = 5 to 15. The columns of these averaged Q matrices, representing admixture proportions for groups discovered in different runs, were then used to define the “K15” grouping. At K = 9, 12, and 13, the Q matrices converged to two different modes according to whether XI-1A or GJ-trop is split (these are labeled as K = 9.1, 12.1 and 13.1).

Group membership for each sample was defined by applying a threshold of 0.65 to admixture components. Samples with no admixture components exceeding 0.65 were classified as follows. If the sum of components for subpopulations within the major groups cA (*circum*-Aus), XI (*Xian*-indica), and GJ (*Geng*-japonica) was ≥0.65, the samples were classified as cA-adm (admixed within cA), XI-adm (admixed within XI) or GJ-adm (admixed within GJ), respectively, and the remaining samples were deemed ‘fully’ admixed. The newly defined groups were mostly align with the previous K = 9 grouping, or were refined and named accordingly (e.g. XI-1B1 and XI-1B2 are two new subgroups within XI-1B).

**Fig. 1:**
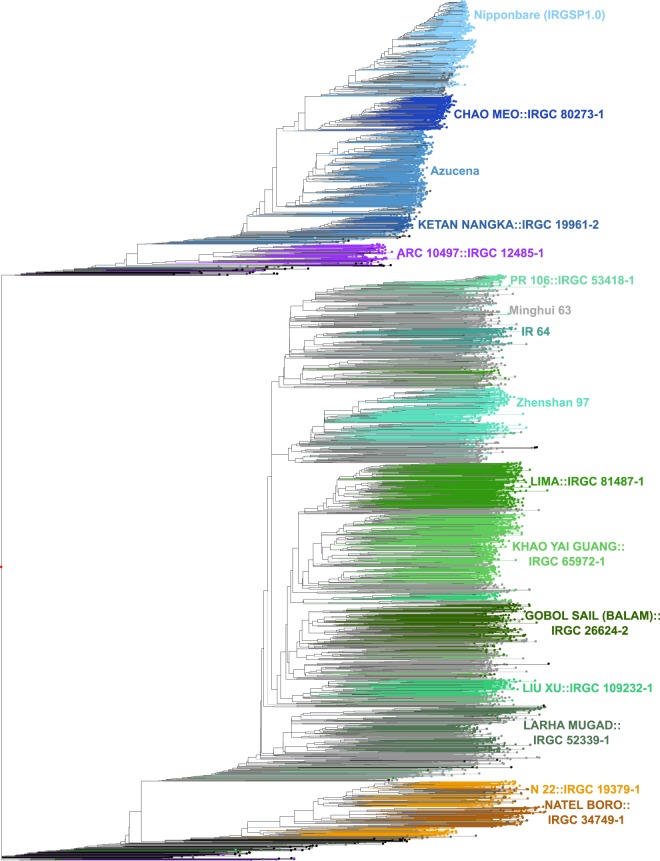
Phylogenetic tree with the accession selected for PSRefSeq sequencing for each of the K = 15 subpopulations and a single admixture group. Groups are colored according to the assignment from Admixture analysis. The subpopulation designation is in parentheses following the name.

The phenogram shown in Fig. 1 was constructed with DARwin v6 (http://darwin.cirad.fr/, unweighted Neighbor-joining) using the identity by state (IBS) distance matrix from Plink on the 4.8 M Filtered SNP set (available at https://snp-seek.irri.org/_download.zul). Colors were assigned to subpopulations based on K15 Admixture results. One entry, MH 63 (XI-adm) represents the admixed types among the XI group.

### Sample selection, collection and nucleic acid preparation

To select accessions to represent the 12 subpopulations of Asian rice that lack high-quality reference genome assemblies, the following strategy was employed. The IBS distance matrix was used for a principal component analysis (PCA) analysis in R to generate 5 component axes. Then, for each of the 12 subpopulations, *i.e. circum-*Aus2 = cA2, *circum*-Basmati = cB, *Geng-*japonica (GJ) subtropical (GJ-subtrp), tropical1 (GJ-trop1) and tropical2 (GJ-trop2), and *Xian-*indica (XI) subpopulations XI-1B1, XI-1B2, XI-2A, XI-2B, XI-3A, XI-3B1, XI-3B2, the centroid of each group in the space spanned by first 5 principal components was determined from the eigenvectors, and the entry closest to the centroid for which seed was available was chosen as the representative for that subpopulation (Table 1).

**Table 1:** Sample collection information for the 12 *Oryza sativa* accessions.

Variety Name	Genetic Stock ID	Country Origin	15 subpops
CHAO MEO::IRGC 80273-1	IRGC 132278	Lao PDR	GJ-subtrp
Azucena	I1A41685	Philippines	GJ-trop1
KETAN NANGKA::IRGC 19961-2	IRGC 128077	Indonesia	GJ-trop2
ARC 10497::IRGC 12485-1	IRGC 117425	India	cB
IR 64	I1A42114	Philippines	XI-1B1
PR 106::IRGC 53418-1	IRGC 127742	India	XI-1B2
LIMA::IRGC 81487-1	IRGC 127564	Indonesia	XI-3A
KHAO YAI GUANG::IRGC 65972-1	IRGC 127518	Thailand	XI-3B1
GOBOL SAIL (BALAM)::IRGC 26624-2	IRGC 132424	Bangladesh	XI-2A
LIU XU::IRGC 109232-1	IRGC 125827	China	XI-3B2
LARHA MUGAD::IRGC 52339-1	IRGC 125619	India	XI-2B
NATEL BORO::IRGC 34749-1	IRGC 127652	Bangladesh	cA2

Subpopulations: GJ = *Geng*-japonica where trop = tropical, subtrp = subtropical; cB = *circum-*Basmati; XI = *Xian*-indica; cA = *circum*-Aus.

Single seed decent (SSD) seed from IR 64 and Azucena were obtained from the Rice Genetics and Genomics Laboratory, CIAT, in Cali, Colombia, and SSD seed from the remaining 10 accessions (Table 1) were obtained from the International Rice Genebank, maintained by IRRI, Los Baños, Philippines. All seed were sown in potting soil and grown under standard greenhouse conditions at UA, Tucson, USA for 6 weeks at which point they were dark treated for 48-hours to reduce starch accumulation. Approximately 20–50 grams of young leaf tissue was then harvested from each accession and immediately flash frozen in liquid nitrogen before being stored at −80 °C prior to DNA extraction. High molecular weight genomic DNA was isolated using a modified CTAB procedure as previously described^18^. The quality of each extraction was checked by pulsed-field electrophoresis (CHEF) on 1% agarose gels for size and restriction enzyme digestibility, and quantified by Qubit fluorometry (Thermo Fisher Scientific, Waltham, MA).

### Library construction and sequencing

Genomic DNA from all 12 accessions were sequenced using the PacBio single-molecule real-time (SMRT) platform, and the Illumina platform for genome size estimations and sequence polishing. High molecular weight (HMW) DNA from each accession was gently sheared into large fragments (*i.e*. 30 Kb–60 Kb) using 26-gauge needles and then end-repaired according to manufacturer’s instructions (Pacific Biosciences). Briefly, using a SMRTbell Express Template Prep Kit, blunt hairpins and sequencing adaptors were ligated to HMW DNA fragments, and DNA sequencing polymerases were bound to the SMRTbell templates. Size selection of large fragments (above 15 Kb) was performed using a BluePippin electrophoresis system (Sage Science). The libraries were quantified using a Qubit Fluorometer (Invitrogen, USA) and the insert mode size was determined using an Agilent fragment analyzer system with sizes ranging between 30 Kb–40 Kb. The libraries then were sequenced using SMRT Cell 1 M chemistry version 3.0 on a PacBio Sequel instrument. The number of long-reads generated per accession ranged from 2.01 million (LIMA::IRGC 81487-1) to 5.40 million (Azucena). The distribution of subreads is shown in Fig. S2 and the average lengths ranged from 10.58 Kb (Azucena) to 20.61 Kb (LIMA::IRGC 81487-1) (Table 2). According to the estimated genome size of the IRGSP RefSeq, the average PacBio sequence coverage for each accession varied from 103x (LIMA::IRGC 81487-1) to 149x (IR 64) (Table 2).

**Table 2:** Sequencing platforms used and data statistics for the 12 *Oryza sativa* genomes.

Variety Name	Sequencing platform	Raw data (Gb)	Depth	Number of subreads (M)	Mean subread length (Kb)
CHAO MEO::IRGC 80273-1	PacBio Sequel	49.1	123×	4.26	11.526
Azucena	PacBio Sequel	57.1	143×	5.40	10.581
KETAN NANGKA::IRGC 19961-2	PacBio Sequel	49.8	125×	2.78	17.876
ARC 10497::IRGC 12485-1	PacBio Sequel	44.7	112×	4.06	11.026
IR 64	PacBio Sequel	59.7	149×	5.24	11.393
PR 106::IRGC 53418-1	PacBio Sequel	42.2	105×	2.08	20.317
LIMA::IRGC 81487-1	PacBio Sequel	41.4	103×	2.01	20.612
KHAO YAI GUANG::IRGC 65972-1	PacBio Sequel	42.5	106×	2.37	17.954
GOBOL SAIL (BALAM)::IRGC 26624-2	PacBio Sequel	42.2	105×	2.13	19.777
LIU XU::IRGC 109232-1	PacBio Sequel	55.3	138×	3.66	15.109
LARHA MUGAD::IRGC 52339-1	PacBio Sequel	45.1	113×	3.22	14.011
NATEL BORO::IRGC 34749-1	PacBio Sequel	44.4	111×	2.74	16.2

For Illumina short-read sequencing, HMW DNA from each accession was sheared to between 250–1000 bp, followed by library construction targeting 350 bp inserts following standard Illumina protocols (San Diego, CA, USA). Each library was 2 × 150 bp paired-end sequenced using an Illumina X-ten platform. Low-quality bases and paired reads with Illumina adaptor sequences were removed using *Trimmomatic*^19^. Quality control for each library data set was carried out with *FastQC*^20^. Finally, between 36.52-Gb and 51.05-Gb of clean data for each accession was generated, and used for genome size estimation (Table S1) by Kmer analysis (Fig. S3) and the Genome Characteristics Estimation (GCE) program^21^.

### Bionano optical genome maps

Bionano optical maps for each accession were generated as previously described^22^, except that ultra-HMW DNA isolation, from approximately 4 g of flash-frozen dark-treated (48 hour) leaf tissue per accession, was performed according to a modified version of the protocol described by Luo and Wing^23^. Prior to labeling, agarose plugs were digested with agarase and the starch and debris removed by short rounds of centrifugation at 13,000 × g. DNA samples were further purified and concentrated by drop dialysis against TE Buffer. Data processing, optical map assembly, hybrid scaffold construction and visualization were performed using the Bionano Solve (Version 3.4) and Bionano Access (v12.5.0) software packages (https://bionanogenomics.com/).

### *De novo* genome assembly

**Fig. 2:**
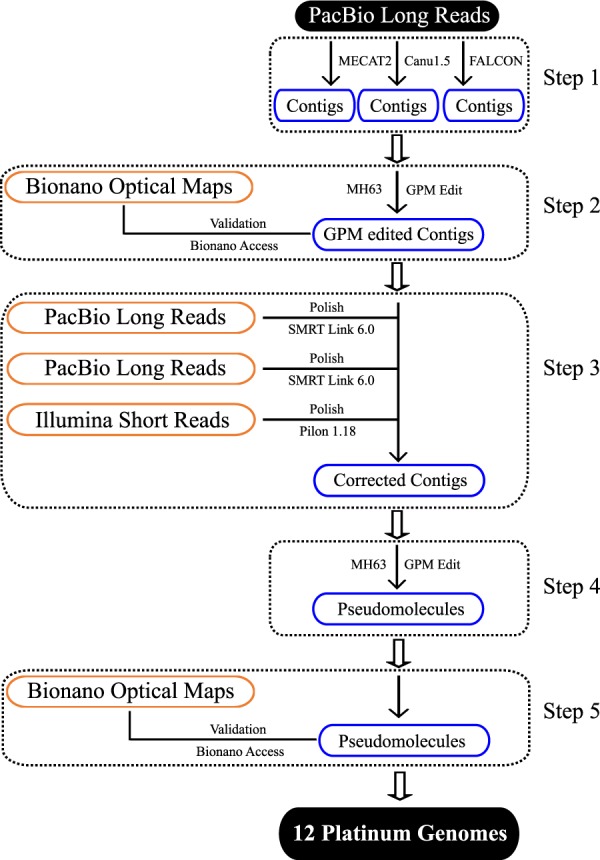
Genome assembly and validation pipeline.

Genome assembly for each of the 12 genomes followed a five-step procedure as shown in (Fig. 2):

Step 1: PacBio subreads were assembled *de novo* into contigs using three genome assembly programs: FALCON^24^, MECAT2^25^ and Canu1.5^26^. The number of *de novo* assembled contigs obtained varied from 51 (e.g. NATEL BORO::IRGC 34749-1 and KETAN NANGKA::IRGC 19961-2) to 1,473 (CHAO MEO::IRGC 80273-1) for the 12 genomes (Table S2).

Step 2: Genome Puzzle Master (GPM) software^27^ was used to merge the *de novo* assembled contigs from the three assemblers, using the high-quality *O. sativa* vg. indica cv. Minghui 63 reference genome sequence (MH63RS2)^13,14^ as a guide. GPM is a semi-automated pipeline that is used to integrate logical relationship data (*i.e*. contigs from three assemblers for each accession) based on a reference guide. Contigs were merged in the ‘assemblyRun’ step, with default parameters (minOverlapSeqToSeq was set at 1 Kb and identitySeqToSeq was set at 99%). Redundant overlapping sequences were also removed for each assembled contig. In addition, we gave contiguous contigs a higher priority than ones with gaps to be retained in each assembly. After manual checking, editing, and redundancy removal, the number of contigs in each assembly ranged from 26 (NATEL BORO::IRGC 34749-1) to 588 (LIU XU::IRGC 109232-1) (Table S2).

Step 3: The sequence quality of each contig was then improved by “sequence polishing”: twice with PacBio long reads and once with Illumina short reads. Briefly, PacBio subreads were aligned to GPM edited contigs using the software *blasr*^28^. All default parameters were used, except minimum align length, which was set to 500 bp. Secondly, the tool *arrow* as implemented in SMRTlink6.0 (Pacific Biosciences of California, Inc) was used for polishing the GPM edited contigs. The *bwa-mem* program^29^ was then used for mapping short Illumina reads onto assembled contigs, and the tool *pilon*^30^ was used for a final polishing step with default settings.

Step 4: The polished contigs for each accession were arranged into pseudomolecules using *GPM*, with MH63RS2^13,14^ as the reference guide. The program *blastn*^31^ with a minimum alignment length of 1 Kb and an e-value < 1e^−5^ as the threshold was used to align the corrected contigs to the reference guide. In doing so, the corrected contigs were assigned to chromosomes, as well as ordered and orientated in the GPM assembly viewer function. The number of contigs after step 4 ranged from a minimum of 15 contigs (GOBOL SAIL (BALAM)::IRGC 26624-2) to a maximum of 104 contigs (IR 64) (Table 3). The assembly size for the 12 accessions ranged from 376.86 Mb (CHAO MEO::IRGC 80273-1) to 393.74 Mb (KHAO YAI GUANG::IRGC 65972-1) (Table 3) and the length of individual chromosome varied from 23.06 Mb (chromosome 9 of CHAO MEO::IRGC 80273-1) to 44.96 Mb (chromosome 1 of LIMA::IRGC 81487-1) (Table S4). The average N50 value was 23.10 Mb, with the highest and the lowest N50 values being 30.91 Mb (LIU XU::IRGC 109232-1) and 7.35 Mb (IR 64), respectively. The average number of gaps among the 12 new genome assemblies was 18, with 8 assemblies containing less than 10 gaps (Table 3).

**Table 3:** *De novo* assembly, BUSCO evaluation and accession numbers in GenBank of the 12 *Oryza sativa* genomes.

Variety Name	BioProject	BioSample	Genome size (bp)	#Contigs	Contig N50 (bp)	#Gaps	Scaffold N50 (bp)	BUSCO	Adjust BUSCO	Genome Accession	SRP	Supplementary Files (Bionano optical map)
CHAO MEO::IRGC 80273-1	PRJNA565484	SAMN12748601	376,856,903	55	11,024,768	43	30,350,168	97.60%	98.49%	VYIH00000000	SRP226088	SUPPF_0000003210
Azucena	PRJNA424001	SAMN08217222	379,627,553	28	22,940,949	16	30,954,872	97.80%	98.69%	PKQC000000000	SRP227255	SUPPF_0000003212
KETAN NANGKA::IRGC 19961-2	PRJNA564615	SAMN12718029	380,759,091	21	22,679,302	9	30,696,581	98.00%	98.89%	VYIC00000000	SRP226080	SUPPF_0000003204
ARC 10497::IRGC 12485-1	PRJNA565479	SAMN12748569	378,463,869	40	17,921,520	28	30,566,713	98.40%	99.30%	VYID00000000	SRP226093	SUPPF_0000003206
IR 64	PRJNA509165	SAMN10564385	386,698,898	104	7,352,909	92	31,218,896	95.70%	96.57%	RWKJ00000000	SRP227298	SUPPF_0000003213
PR 106::IRGC 53418-1	PRJNA563359	SAMN12672924	391,176,105	16	27,051,416	4	32,028,703	96.60%	97.48%	VYIB00000000	SRP226078	SUPPF_0000003202
LIMA::IRGC 81487-1	PRJNA564572	SAMN12715984	392,625,308	17	27,369,091	5	32,421,942	98.50%	99.40%	VXJH00000000	SRP226079	SUPPF_0000003203
KHAO YAI GUANG::IRGC 65972-1	PRJNA565481	SAMN12748590	393,737,720	19	21,823,919	7	32,080,718	98.60%	99.50%	VYIF00000000	SRP226086	SUPPF_0000003208
GOBOL SAIL (BALAM)::IRGC 26624-2	PRJNA564763	SAMN12721963	391,772,995	15	29,604,901	3	31,753,752	97.90%	98.79%	VXJI00000000	SRP226082	SUPPF_0000003205
LIU XU::IRGC 109232-1	PRJNA577228	SAMN13021815	392,033,263	17	30,913,760	5	32,301,089	98.40%	99.30%	WGGU00000000	SRP226085	SUPPF_0000003211
LARHA MUGAD::IRGC 52339-1	PRJNA565480	SAMN12748589	390,195,943	16	30,747,645	4	32,107,744	98.60%	99.50%	VYIE00000000	SRP226084	SUPPF_0000003207
NATEL BORO::IRGC 34749-1	PRJNA565483	SAMN12748600	383,720,936	16	27,825,079	4	31,305,988	98.10%	98.99%	VYIG00000000	SRP226087	SUPPF_0000003209

**Fig. 3:**
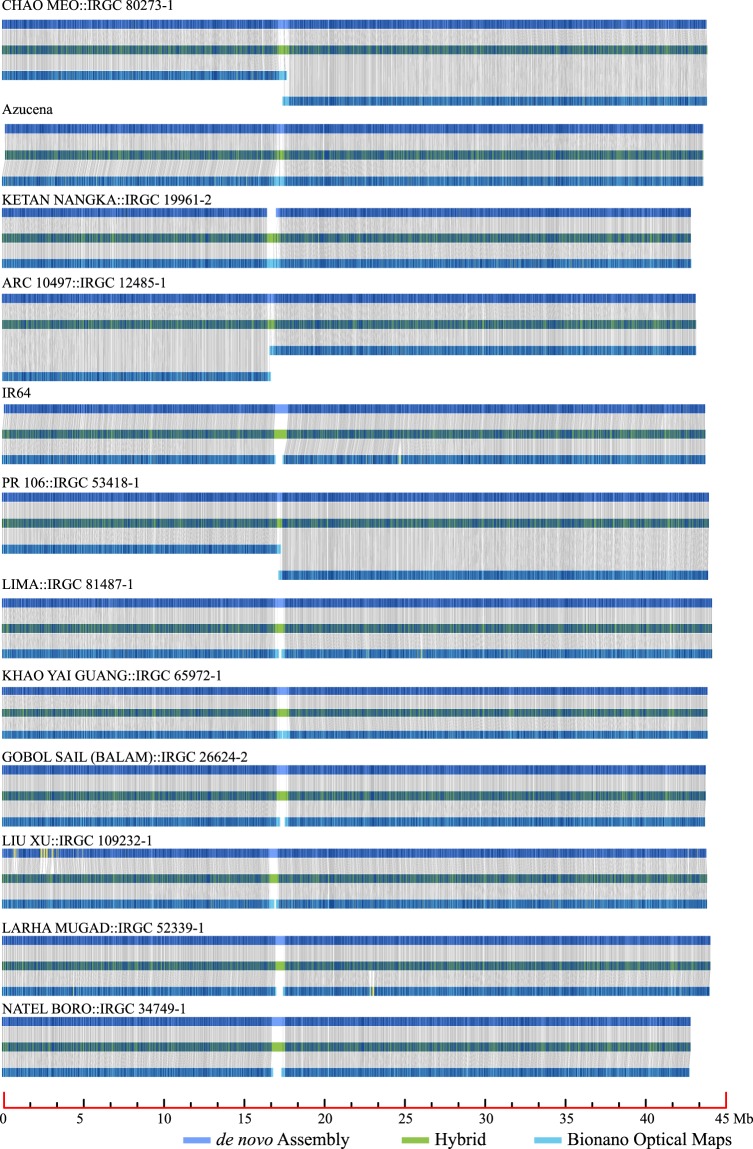
Bionano optical map validation of chromosome 1 for 12 *de novo* assemblies.

Step 5: To independently validate our assemblies, we generated and compared Bionano optical maps to each assembly. In total, 17 (Azucena) to 56 (LIU XU::IRGC 109232-1) Bionano optical contigs were constructed for all 12 rice accessions, which yielded contig N50 values of between 22.75 Mb (CHAO MEO::IRGC 80273-1) to 31.45 Mb (KHAO YAI GUANG::IRGC 65972-1) (Table S5). As shown in Figs. 3 and S4, the chromosomes and/or chromosome arms of all 12 *de novo* assemblies were highly supported by these ultra-long optical maps. Although rare, a few discrepancies between the optical maps and genome assemblies could be found and are likely due to small errors and chimeras that were produced through both the optical map and sequence assembly pipelines^15^.

Following these five steps, we were able to produce 12 near-gap-free *Oryza sativa* platinum standard reference genome sequences (PSRefSeqs) that represent 12 of 15 subpopulations of cultivated Asian rice.

### BUSCO evaluation

The Benchmarking Universal Single-Copy Orthologs (BUSCO3.0) software package^32^ was employed to evaluate the gene space completeness of the 12 genome assemblies. These genomes captured, on average, 97.9% of the BUSCO reference gene set, with a minimum of 95.7% (IR64) and a maximum of 98.6% (LARHA MUGAD::IRGC 52339-1 and KHAO YAI GUANG::IRGC 65972-1) (Table 3).

Of note, when performing this analysis, we observed that on average 30 out of the 1,440 conserved BUSCO genes tested (https://www.orthodb.org/v9/index.html) were missing from each new assembly, 16 of which were not present in all 12, plus the IRGSP RefSeq-1.0, ZS 97, MH 63 and N 22 RefSeqs (Fig. S5). This result suggested that these 16 “conserved” genes may not exist in rice, or other cereal genomes, thereby artificially reducing the BUSCO gene space scores for our 12 assemblies. To test this hypothesis, we searched for all 16 genes missing in maize, which diverged from rice about 50 million years ago (MYA)^33–35^. We found that 13 of the 16 genes in question could not be found in 3 high-quality recently published maize genome assemblies (Fig. S5) and therefore, concluded that 13 of the 16 “conserved” genes in the BUSCO database are not present in cereals, and should be excluded from our gene space analysis. Taking this into account, we recalculated the BUSCO gene space content for each of 12 assemblies and found that 10 of 12 assemblies captured more than 98% of the BUSCO gene set (Table 3).

### Transposable element (TE) prediction

To determine the pan-transposable element content of cultivated Asian rice, we analyzed the 12 new reference genomes, presented here, along with the MH 63, ZS 97, N 22 PacBio reference genomes. In addition, we also included a reanalysis of the IRGSP RefSeq-1.0, as it is conventionally considered the standard rice genome for which all comparisons are conducted.

A search for sequences similar to TEs was carried out using RepeatMasker^36^, run under default parameters with the exception of the option: -no_is –nolow, and that an updated in-house version of the publicly available MSU_6.9.5 library^37^, retrieved from https://github.com/oushujun/EDTA/blob/master/database/Rice_MSU7.fasta.std6.9.5.out, called “rice 7.0.0.liban” was used. The average TE content of this 16 genome data set was 47.66% with a minimum value of 46.07% in IRGSP RefSeq-1.0 and a maximum of 48.27% in KHAO YAI GUANG::IRGC 65972-1 (Table 4). The major contribution to this fraction was composed of long terminal repeat retrotransposons (LTR-RTs, min: 23.55%, max: 27.27% and average: 25.96%) followed by DNA-TEs (min:14.87%, max, 16.18% and average: 15.26%). Long interspersed nuclear elements (LINEs) and short interspersed nuclear elements (SINEs) were identified as on average 1.43% and 0.39% of the 16 genomes, respectively.

**Table 4:** Abundance of the major TE classes in the 16 *Oryza sativa* genomes.

Variety Name	Total	LTR-RT	LINEs	SINEs	DNA_TEs	Unclassified
NIPPONBARE	46.07	23.55	1.52	0.41	16.18	4.41
CHAO MEO::IRGC 80273-1	46.25	24.00	1.46	0.40	15.59	4.80
Azucena	47.07	24.48	1.47	0.40	15.82	4.89
KETAN NANGKA::IRGC 19961-2	46.99	24.87	1.47	0.40	15.72	4.53
ARC 10497::IRGC 12485-1	46.95	24.74	1.48	0.40	15.68	4.65
PR 106::IRGC 53418-1	47.95	26.82	1.41	0.39	15.05	4.28
Minghui 63	47.97	26.61	1.44	0.4	15.3	4.22
IR 64	47.87	26.82	1.42	0.40	14.97	4.26
Zhenshan 97	47.95	26.79	1.42	0.39	15.19	4.16
LIMA::IRGC 81487-1	48.04	26.87	1.40	0.39	15.01	4.37
KHAO YAI GUANG::IRGC 65972-1	48.27	27.27	1.40	0.39	14.87	4.34
GOBOL SAIL (BALAM)::IRGC 26624-2	48.15	26.99	1.40	0.39	14.99	4.38
LIU XU::IRGC 109232-1	46.92	27.06	1.26	0.32	14.31	3.97
LARHA MUGAD::IRGC 52339-1	48.05	26.74	1.41	0.39	15.09	4.42
N 22::IRGC 19379-1	47.79	25.95	1.44	0.39	15.20	4.81
NATEL BORO::IRGC 34749-1	47.33	25.75	1.42	0.40	15.12	4.64

### Structural Variants

Each genome assembly, as described above, was fragmented using the EMBOSS tool *splitter*^38^ to create a 10x genome equivalent redundant set of 50 kb reads. These reads were then mapped onto every other genome assembly using the tool *NGMLR*^39^. Finally, the software *SVIM*^40^ was run under default parameters to parse the mapping output. Only insertions, deletions and tandem duplications up to a maximum length of 25 Kb were considered in this analysis.

The results of this analysis identified several thousand insertions and deletions whenever an assembly was compared to any other. Greater variability was found between varieties belonging to different major groups (e.g. *Geng-*japonica [*GJ*] vs. *Xian*-indica [*XI*] than occurred between those within these groups. The amount of genome sequences with structural variation between any two varieties ranged from 17.57 Mb to 41.54 Mb for those belonging to the indica (XI) varietal group (avg: 31.75 Mb) and from 18.55 Mb to 23.07 Mb (avg: 21.00 Mb) for those in the japonica (GJ) varietal group. When all 16 genomes are considered together, the range is between 17.57 Mb and 41.54 Mb, with an average value of 33.70 Mb (Table S6). The total unshared fraction collected out of all pairwise comparisons was composed for 89.89% by TE related sequences.

## Data Records

Data for all 12 genome shotgun sequencing projects have been deposited in Genbank (https://www.ncbi.nlm.nih.gov/) including PacBio and Illumina raw data^41–52^, the twelve reference genome assemblies^53–64^ and Bionano optical maps. BioProjects, BioSamples, Genome assemblies, Sequence Read Archives (SRA) accession numbers and supplementary files (*i.e*. Bionano optical maps) of the 12 new assemblies are listed in Table 3. Transposable element, structural variation annotations and also the Bionano optical maps are available in the figshare link (https://figshare.com/s/dcdaea3adae5c44e2e31)^65^.

## Technical Validation

### DNA sample quality

DNA quality was checked by pulsed-field gel electrophoresis for size and restriction enzyme digestibility. Nucleic acid concentrations were quantified by Qubit fluorometry (Thermo Fisher Scientific, Waltham, MA).

### Illumina libraries

Illumina libraries were quantified by qPCR using the KAPA Library Quantification Kit for Illumina Libraries (KapaBiosystems, Wilmington, MA, USA), and library profiles were evaluated with an Agilent 2100 Bioanalyzer (Agilent Technologies, Santa Clara, CA, USA).

### Gene space completeness

Benchmarking Universal Single-Copy Orthologs (BUSCO3.0) was executed using the embryophyta_odb9.tar.gz database to assess the gene space of each genome, minus 13 genes that do not appear to exist in the cereal genomes tested (Fig. S5).

### Assembly accuracy

Bionano optical maps were generated and used to validate all 12 genome assemblies.

## Supplementary information

Supplementary information

## Data Availability

The population re-analysis of 3K-RG dataset and 12 genome assemblies were obtained using several publicly available software packages. To allow researchers to precisely repeat any steps, the settings and the parameters used are provided below: Population structure: ADMIXTURE was run with default options. The R scripts for further population structure analysis, including setting up CLUMPP files, can be found in Github repository https://github.com/dchebotarov/Q-aggr. Genome size estimation: The K-mer and GCE program were employed for genome size estimation. Command line: kmer_freq_hash -k (13-17) -l genome.list -a 10 -d 10 -t 8 -i 400000000 -o 0 -p genom_kmer(13-17) & > genome_kmer(13-17)_freq.log, and gce -f genom _kmer(13-17).freq.stat -c $peak -g #amount -m 1 -D 8 -b 1 -H 1 > genome.Table 2 > genom_kmer(13-17).log Genome assembly: (1) *MECAT2*: all parameters were set to the defaults. Command line: mecat.pl config_file.txt, mecat.pl correct config_file.txt and mecat.pl assemble config_file.txt (2) *Canu1.5*: all parameters were set to the defaults. Command line: canu -d canu -p canu genomeSize = 400 m -pacbio-raw rawreads.fasta (3) *FALCON*: all parameters were set to the defaults. Command line: fc_run.py fc_run.cfg & > fc_run.out (4) *GPM*: manual edit with merging *de novo* assemblies from *MECAT2*, *Canu1*.*5*, and *FALCON* Polishing: (1) *arrow*: all parameters were set to the defaults except alignment length = 500 bp. The *arrow* polish was carried out by the SMRT Link v6.0 webpage (https://www.pacb.com/support/software-downloads/). (2) *pilon1.18*: all parameters were set to the defaults. BUSCO: The BUSCO3.0 version was employed in this study. Command line: run_BUSCO.py -i genome.fasta -o genome -l embryophyta_odb9 -m genome -c 16 RepeatMasker: The repeat sequences were employed with the library rice7.0.0_liban in-house. Command line: RepeatMasker -pa 24 -x -no_is -nolow -cutoff 250 -lib rice7.0.0.liban.txt genome.fasta

## References

[1] Seck P-A, Diagne A, Mohanty S, Wopereis M-C (2012). Crops that feed the world 7: Rice. Food security.

[2] Merrey, D.-J. *et al*. Agricultural Development and Sustainable Intensification. *Routledge* (2018).

[3] Wing A-R, Michael D-P, Zhang Q-F (2018). The rice genome revolution: from an ancient grain to Green Super Rice. Nature Reviews Genetics.

[4] 3K RGP (2014). The 3,000 rice genomes project. GigaScience.

[5] Stein J-C (2018). Genomes of 13 domesticated and wild rice relatives highlight genetic conservation, turnover and innovation across the genus Oryza. Nature genetics.

[6] Kawahara Y (2013). Improvement of the Oryza sativa Nipponbare reference genome using next generation sequence and optical map data. Rice.

[7] International Rice Genome Sequencing Project (2005). The map-based sequence of the rice genome. Nature.

[8] Thomson M-J (2017). Large-scale deployment of a rice 6 K SNP array for genetics and breeding applications. Rice.

[9] McNally K-L (2009). Genomewide SNP variation reveals relationships among landraces and modern varieties of rice. Proceedings of the National Academy of Sciences.

[10] Huang X-H (2012). A map of rice genome variation reveals the origin of cultivated rice. Nature.

[11] Zhao Q (2018). Pan-genome analysis highlights the extent of genomic variation in cultivated and wild rice. Nature genetics.

[12] Wang W (2018). Genomic variation in 3,010 diverse accessions of Asian cultivated rice. Nature.

[13] Zhang J (2016). Building two indica rice reference genomes with PacBio long-read and Illumina paired-end sequencing data. Scientific data..

[14] Zhang J (2016). Extensive sequence divergence between the reference genomes of two elite indica rice varieties Zhenshan 97 and Minghui 63. Proc. Natl. Acad. Sci..

[15] Udall J-A, Kelly D (2018). Is it ordered correctly? Validating genome assemblies by optical mapping. The Plant Cell.

[16] Alexander DH, Novembre J, Lange K (2009). Fast model-based estimation of ancestry in unrelated individuals. Genome research.

[17] Jakobsson M, Noah AR (2007). CLUMPP: a cluster matching and permutation program for dealing with label switching and multimodality in analysis of population structure. Bioinformatics.

[18] Porebski S, Bailey L-G, Baum B-R (1997). Modification of a CTAB DNA extraction protocol for plants containing high polysaccharide and polyphenol components. Plant molecular biology reporter.

[19] Bolger A-M, Marc L, Bjoern U (2014). Trimmomatic: a flexible trimmer for Illumina sequence data. Bioinformatics.

[20] Brown J, Meg P, Lee AM (2017). FQC Dashboard: integrates FastQC results into a web-based, interactive, and extensible FASTQ quality control tool. Bioinformatics.

[21] Liu, B. *et al*. Estimation of genomic characteristics by analyzing k-mer frequency in *de novo* genome projects. Preprint at, https://arxiv.org/abs/1308.2012 (2013).

[22] Ou, S. *et al*. Effect of sequence depth and length in long-read assembly of the maize inbred nc358. Preprint at, 10.1101/858365v2.full (2019). 10.1038/s41467-020-16037-7 PMC7211024 32385271

[23] Luo M, Wing A-R (2003). An improved method for plant BAC library construction. Plant functional genomics. Humana Press.

[24] Chin C (2016). Phased diploid genome assembly with single-molecule real-time sequencing. Nature methods.

[25] Xiao C (2017). MECAT: fast mapping, error correction, and de novo assembly for single-molecule sequencing reads. nature methods.

[26] Koren S (2017). Canu: scalable and accurate long-read assembly via adaptive k-mer weighting and repeat separation. Genome research.

[27] Zhang J (2016). Genome puzzle master (GPM): an integrated pipeline for building and editing pseudomolecules from fragmented sequences. Bioinformatics.

[28] Chaisson M-J, Tesler G (2012). Mapping single molecule sequencing reads using basic local alignment with successive refinement (BLASR): application and theory. BMC bioinformatics.

[29] Li, H. Aligning sequence reads, clone sequences and assembly contigs with BWA-MEM. Preprint at, https://arxiv.org/abs/1303.3997 (2013).

[30] Walker B-J (2014). Pilon: an integrated tool for comprehensive microbial variant detection and genome assembly improvement. Plos One.

[31] Altschul S-F (1997). Gapped BLAST and PSI-BLAST: a new generation of protein database search programs. Nucleic acids research.

[32] Simão F-A (2015). BUSCO: assessing genome assembly and annotation completeness with single-copy orthologs. Bioinformatics.

[33] Wolfe K-H (1989). Date of the monocot-dicot divergence estimated from chloroplast DNA sequence data. Proceedings of the National Academy of Sciences.

[34] Gale M-D, Katrien MD (1998). Comparative genetics in the grasses. Proceedings of the National Academy of Sciences.

[35] Guo H (2019). Gene duplication and genetic innovation in cereal genomes. Genome research.

[36] Maja T, Chen N (2009). Using RepeatMasker to identify repetitive elements in genomic sequences. Current protocols in bioinformatics.

[37] Ou S-J (2019). Benchmarking transposable element annotation methods for creation of a streamlined, comprehensive pipeline. Genome Biology.

[38] Rice P, Ian L, Alan B (2000). EMBOSS: the European molecular biology open software suite. Trends in Genetics.

[39] Sedlazeck F-J (2018). Accurate detection of complex structural variations using single-molecule sequencing. Nature methods.

[40] Heller D, Martin V (2019). SVIM: structural variant identification using mapped long reads. Bioinformatics.

[41] (2019). NCBI Sequence Read Archive.

[42] (2019). NCBI Sequence Read Archive.

[43] (2019). NCBI Sequence Read Archive.

[44] (2019). NCBI Sequence Read Archive.

[45] (2019). NCBI Sequence Read Archive.

[46] (2019). NCBI Sequence Read Archive.

[47] (2019). NCBI Sequence Read Archive.

[48] (2019). NCBI Sequence Read Archive.

[49] (2019). NCBI Sequence Read Archive.

[50] (2019). NCBI Sequence Read Archive.

[51] (2019). NCBI Sequence Read Archive.

[52] (2019). NCBI Sequence Read Archive.

[53] Zhang J (2019). GenBank.

[54] Zhang J (2019). GenBank.

[55] Zhou Y (2019). GenBank.

[56] Zhou Y (2019). GenBank.

[57] Zhou Y (2019). GenBank.

[58] Zhou Y (2019). GenBank.

[59] Zhou Y (2019). GenBank.

[60] Zhou Y (2019). GenBank.

[61] Zhou Y (2019). GenBank.

[62] Zhou Y (2019). GenBank.

[63] Zhou Y (2019). GenBank.

[64] Zhou Y (2019). GenBank.

[65] Zhou Y (2020). figshare.

