# Supplementary material for: A platinum standard pan-genome resource that represents the population structure of Asian rice
Source: Sci Data. 2020 Apr 7;7:113. doi: 10.1038/s41597-020-0438-2 (PMC7138821; doi:10.1038/s41597-020-0438-2)

Supplementary Information

Supplementary Tables

Supplementary Table 1. Summary of Illumina genome survey sequences for 12 *Oryza sativa* genomes.

Supplementary Table 2. Genome features of *de novo* assemblies for 12 *Oryza sativa* accessions by Canu1.5, FALCON and MECAT2.

Supplementary Table 3. Genome features of 12 *Oryza sativa* accessions by GPM editing.

Supplementary Table 4. Chromosome length (Mb) of 12 *Oryza sativa* genomes.

Supplementary Table 5. Bionano optical map statistics of 12 *Oryza sativa* genomes.

Supplementary Table 6. Summary of large structural variation (50-25,000 bp) by comparison of each of 16 genomes to every other genome (including 12 genomes from this study and 4 previously reported: MH63, ZS97, N 22 and the IRGSP RefSeq).

Supplementary Figures

Supplementary Figure 1. Admixture results for K=5 to 15. The samples are grouped according to the new classification. At K=9,12,13, the Q matrices converged to two different modes, differing according to whether ind1A is split, or tropical japonica.

Supplementary Figure 2. Length distribution of PacBio long reads used for 12 *Oryza sativa* genome assemblies.

Supplementary Figure 3. K-mer analysis of Illumina short sequences that were used for genome size estimation with the GCE program.

Supplementary Figure 4. Bionano Access visualization view for 12 *de novo* assemblies with Bionano optical maps and their underlying alignments.

Supplementary Figure 5. Summary of missing genes in the BUSCO gene space evaluation of 12 *de novo Oryza sativa* assemblies, 4 public *Oryza sativa* PSRefSeqs and 3 high-quality *Zea mays* genomes.

Supplementary Table 1.

Variety Name	Illumina Total (bp)	Genome Size (bp) Kmer = 14	Illumina Depth (X)
CHAO MEO::IRGC 80273-1	38,600,557,632	396,965,000	97.24
Azucena	13,506,028,120	390,284,000	34.61
KETAN NANGKA::IRGC 19961-2	51,046,107,492	393,783,000	97.59
ARC 10497::IRGC 12485-1	42,487,266,828	411,065,000	103.36
IR 64	11,089,683,636	378,699,000	29.28
PR 106::IRGC 53418-1	41,312,634,506	427,654,000	96.60
LIMA::IRGC 81487-1	44,393,932,352	427,905,000	103.75
KHAO YAI GUANG::IRGC 65972-1	36,515,938,608	452,866,000	80.63
GOBOL SAIL (BALAM)::IRGC 26624-2	35,404,680,382	414,841,000	85.35
LIU XU::IRGC 109232-1	47,436,101,266	420,565,000	112.79
LARHA MUGAD::IRGC 52339-1	44,349,661,870	404,289,000	109.70
NATEL BORO::IRGC 34749-1	43,746,343,786	372,517,000	117.43

Supplementary Table 2.

Assemblers	Fetures	CHAO MEO::IRGC 80273-1	Azucena	KETAN NANGKA::IRGC 19961-2	ARC 10497::IRGC 12485-1	IR 64	PR 106::IRGC 53418-1	LIMA::IRGC 81487-1	KHAO YAI GUANG::IRGC 65972-1	GOBOL SAIL (BALAM)::IRGC 26624-2	LIU XU::IRGC 109232-1	LARHA MUGAD::IRGC 52339-1	NATEL BORO::IRGC 34749-1
	Number of sequences	152	1432	51	111	542	73	137	88	77	758	144	51
	Total size of sequences	385,300,312 bp.	426,854,248 bp.	384,182,251 bp.	382,632,343 bp.	450,675,276 bp.	394,777,416 bp.	406,108,421 bp.	398,327,588 bp.	394,382,542 bp.	496,174,224 bp.	396,786,868 bp.	381,520,998 bp.
	Longest sequence	19,303,668 bp.	13,316,004 bp.	29,546,610 bp.	26,399,646 bp.	11,833,599 bp.	31,338,188 bp.	27,196,387 bp.	32,065,732 bp.	29,601,517 bp.	38,163,182 bp.	32,133,799 bp.	31,694,912 bp.
	Shortest sequence	31,263 bp.	53 bp.	33,228 bp.	25,840 bp.	4,470 bp.	71,365 bp.	60,302 bp.	62,199 bp.	56,816 bp.	30,651 bp.	5,557 bp.	42,889 bp.
	Number of sequences > 500 nt	152 (385,300,312 bp).	1428 (426,853,447 bp).	51 (384,182,251 bp).	111 (382,632,343 bp).	542 (450,675,276 bp).	73 (394,777,416 bp).	137 (406,108,421 bp).	88 (398,327,588 bp).	77 (394,382,542 bp).	758 (496,174,224 bp).	144 (396,786,868 bp).	51 (381,520,998 bp).
	Number of sequences > 1k nt	152 (385,300,312 bp).	1421 (426,847,636 bp).	51 (384,182,251 bp).	111 (382,632,343 bp).	542 (450,675,276 bp).	73 (394,777,416 bp).	137 (406,108,421 bp).	88 (398,327,588 bp).	77 (394,382,542 bp).	758 (496,174,224 bp).	144 (396,786,868 bp).	51 (381,520,998 bp).
canu	Number of sequences > 10k nt	152 (385,300,312 bp).	1247 (425,796,067 bp).	51 (384,182,251 bp).	111 (382,632,343 bp).	541 (450,670,806 bp).	73 (394,777,416 bp).	137 (406,108,421 bp).	88 (398,327,588 bp).	77 (394,382,542 bp).	758 (496,174,224 bp).	142 (396,775,025 bp).	51 (381,520,998 bp).
	Number of sequences > 100k nt	95 (381,554,518 bp).	204 (385,954,685 bp).	43 (383,650,997 bp).	69 (379,988,479 bp).	274 (432,659,071 bp).	61 (393,767,794 bp).	111 (403,956,780 bp).	74 (397,211,223 bp).	68 (393,677,326 bp).	541 (478,029,256 bp).	59 (391,718,277 bp).	35 (380,362,503 bp).
	Number of sequences > 1M nt	53 (370,549,910 bp).	96 (352,401,999 bp).	27 (380,102,219 bp).	51 (376,259,174 bp).	102 (387,243,850 bp).	27 (387,700,820 bp).	35 (387,829,465 bp).	35 (389,640,858 bp).	34 (385,829,226 bp).	70 (371,155,003 bp).	35 (385,063,650 bp).	24 (378,301,198 bp).
	Mean sequence length	2,534,870.00	298,082.00	7,532,985.00	3,447,138.00	831,504.00	5,407,909.00	2,964,295.00	4,526,449.00	5,121,851.00	654,583.00	2,755,464.00	7,480,803.00
	Median sequence length	154,148.5 bp.	33,929.5 bp.	3,371,535 bp.	260,726 bp.	101,136.5 bp.	170,322 bp.	163,678 bp.	195,127 bp.	401,049 bp.	137,828.5 bp.	82,020 bp.	317,268 bp.
	N50 sequence length	9,716,915 bp.	3,888,558 bp.	17,707,699 bp.	9,934,016 bp.	3,959,778 bp.	20,999,472 bp.	14,262,161 bp.	17,266,774 bp.	15,685,983 bp.	5,977,894 bp.	17,747,777 bp.	21,678,103 bp.
	L50 sequence count	15	35	9	12	33	8	11	10	10	18	8	7
	Number of sequences	1,473.00	104	447	1,484.00	1226	681	647	586	603	749	712	539
	Total size of sequences	417,964,237 bp.	384,739,691 bp.	397,017,154 bp.	417,858,139 bp.	462,958,049 bp.	424,682,512 bp.	425,971,071 bp.	415,255,023 bp.	418,006,874 bp.	463,520,710 bp.	418,962,941 bp.	403,797,739 bp.
	Longest sequence	6,043,249 bp.	31,965,826 bp.	17,654,262 bp.	6,506,933 bp.	6,461,080 bp.	18,987,500 bp.	21,189,681 bp.	15,630,036 bp.	25,154,512 bp.	10,984,374 bp.	21,196,415 bp.	19,949,698 bp.
	Shortest sequence	243 bp.	1,510 bp.	1,305 bp.	340 bp.	367 bp.	1,077 bp.	307 bp.	43 bp.	1,403 bp.	0 bp.	362 bp.	733 bp.
	Number of sequences > 500 nt	1472 (417,963,994 bp).	104 (384,739,691 bp).	447 (397,017,154 bp).	1483 (417,857,799 bp).	1223 (462,956,743 bp).	681 (424,682,512 bp).	645 (425,970,351 bp).	585 (415,254,980 bp).	603 (418,006,874 bp).	742 (463,520,710 bp).	710 (418,962,103 bp).	539 (403,797,739 bp).
	Number of sequences > 1k nt	1471 (417,963,457 bp).	104 (384,739,691 bp).	447 (397,017,154 bp).	1483 (417,857,799 bp).	1221 (462,955,381 bp).	681 (424,682,512 bp).	643 (425,968,941 bp).	583 (415,253,603 bp).	603 (418,006,874 bp).	742 (463,520,710 bp).	707 (418,959,311 bp).	536 (403,795,287 bp).
falcon	Number of sequences > 10k nt	1383 (417,384,090 bp).	103 (384,738,181 bp).	405 (396,770,358 bp).	1381 (417,198,008 bp).	1137 (462,474,043 bp).	642 (424,517,345 bp).	599 (425,701,310 bp).	537 (414,986,546 bp).	553 (417,734,033 bp).	742 (463,520,710 bp).	620 (418,418,022 bp).	492 (403,553,154 bp).
	Number of sequences > 100k nt	383 (377,807,156 bp).	58 (382,027,799 bp).	109 (382,922,790 bp).	413 (379,790,675 bp).	362 (428,819,002 bp).	170 (402,714,933 bp).	157 (403,790,785 bp).	151 (397,535,932 bp).	130 (397,593,735 bp).	382 (444,176,145 bp).	166 (398,730,047 bp).	105 (384,697,784 bp).
	Number of sequences > 1M nt	130 (277,224,326 bp).	39 (378,370,621 bp).	70 (370,631,657 bp).	134 (272,722,546 bp).	143 (356,145,432 bp).	71 (383,112,141 bp).	79 (386,015,143 bp).	91 (373,127,562 bp).	64 (379,068,674 bp).	106 (352,709,101 bp).	83 (369,447,989 bp).	66 (371,577,588 bp).
	Mean sequence length	283,750.00	3,699,420.00	888,181.00	281,575.00	377,616.00	623,616.00	658,378.00	708,626.00	693,212.00	618,852.00	588,431.00	749,160.00
	Median sequence length	42,705 bp.	121,616 bp.	51,927 bp.	40,515 bp.	51,650 bp.	52,979 bp.	55,976 bp.	52,379 bp.	50,373 bp.	102,363 bp.	47,354.5 bp.	56,704 bp.
	N50 sequence length	1,616,332 bp.	16,106,380 bp.	6,802,876 bp.	1,564,692 bp.	2,200,738 bp.	7,973,901 bp.	7,470,691 bp.	4,696,015 bp.	7,651,249 bp.	3,265,125 bp.	5,662,344 bp.	8,020,714 bp.
	L50 sequence count	78	9	18	84	60	19	19	28	17	40	25	16
	Number of sequences	241	116	97	230	501	113	143	116	64	679	193	201
	Total size of sequences	375,395,687 bp.	378,046,431 bp.	382,778,126 bp.	377,640,874 bp.	418,790,768 bp.	395,404,167 bp.	400,608,885 bp.	367,073,907 bp.	393,211,896 bp.	446,168,466 bp.	393,508,832 bp.	385,696,147 bp.
	Longest sequence	10,940,410 bp.	23,392,999 bp.	29,487,693 bp.	16,830,803 bp.	9,620,289 bp.	34,489,834 bp.	36,848,924 bp.	29,024,776 bp.	44,304,478 bp.	38,493,727 bp.	29,810,963 bp.	31,845,677 bp.
	Shortest sequence	610 bp.	1,219 bp.	1,074 bp.	549 bp.	28,131 bp.	1,339 bp.	1,339 bp.	1,598 bp.	4,693 bp.	549 bp.	664 bp.	691 bp.
	Number of sequences > 500 nt	241 (375,395,687 bp).	116 (378,046,431 bp).	97 (382,778,126 bp).	230 (377,640,874 bp).	501 (418,790,768 bp).	113 (395,404,167 bp).	143 (400,608,885 bp).	116 (367,073,907 bp).	64 (393,211,896 bp).	679 (446,168,466 bp).	193 (393,508,832 bp).	201 (385,696,147 bp).
	Number of sequences > 1k nt	238 (375,393,190 bp).	116 (378,046,431 bp).	97 (382,778,126 bp).	226 (377,637,666 bp).	501 (418,790,768 bp).	113 (395,404,167 bp).	143 (400,608,885 bp).	116 (367,073,907 bp).	64 (393,211,896 bp).	668 (446,160,455 bp).	192 (393,508,168 bp).	198 (385,693,696 bp).
mecat2	Number of sequences > 10k nt	217 (375,281,276 bp).	106 (377,989,173 bp).	83 (382,698,180 bp).	194 (377,488,933 bp).	501 (418,790,768 bp).	99 (395,330,632 bp).	117 (400,468,309 bp).	107 (367,032,103 bp).	60 (393,184,910 bp).	479 (445,139,776 bp).	166 (393,377,173 bp).	143 (385,407,723 bp).
	Number of sequences > 100k nt	136 (372,059,148 bp).	67 (376,358,737 bp).	37 (380,556,280 bp).	129 (374,884,549 bp).	318 (406,303,185 bp).	34 (392,932,928 bp).	50 (397,830,979 bp).	36 (363,770,126 bp).	33 (391,657,293 bp).	220 (436,276,969 bp).	62 (388,790,002 bp).	37 (380,996,815 bp).
	Number of sequences > 1M nt	81 (341,969,740 bp).	51 (368,967,817 bp).	29 (378,617,893 bp).	90 (357,474,872 bp).	122 (331,039,223 bp).	24 (389,615,790 bp).	33 (392,934,938 bp).	25 (359,778,489 bp).	22 (389,148,818 bp).	87 (385,649,883 bp).	47 (385,427,209 bp).	25 (379,394,694 bp).
	Mean sequence length	1,557,658.00	3,259,020.00	3,946,166.00	1,641,916.00	835,909.00	3,499,151.00	2,801,460.00	3,164,430.00	6,143,935.00	657,096.00	2,038,905.00	1,918,886.00
	Median sequence length	363,408 bp.	504,521 bp.	64,332 bp.	317,997.5 bp.	190,677 bp.	39,878 bp.	46,818 bp.	58,138 bp.	116,075.5 bp.	30,962 bp.	50,966 bp.	29,747 bp.
	N50 sequence length	5,336,915 bp.	10,440,731 bp.	17,584,371 bp.	5,089,065 bp.	2,737,939 bp.	22,469,819 bp.	17,639,931 bp.	17,840,582 bp.	20,803,229 bp.	6,899,863 bp.	12,298,593 bp.	18,402,708 bp.
	L50 sequence count	25	13	9	22	46	8	9	8	7	16	12	8

Supplementary Table 3.

Fetures	CHAO MEO::IRGC 80273-1	Azucena	KETAN NANGKA::IRGC 19961-2	ARC 10497::IRGC 12485-1	IR 64	PR 106::IRGC 53418-1	LIMA::IRGC 81487-1	KHAO YAI GUANG::IRGC 65972-1	GOBOL SAIL (BALAM)::IRGC 26624-2	LIU XU::IRGC 109232-1	LARHA MUGAD::IRGC 52339-1	NATEL BORO::IRGC 34749-1
Number of sequences	136	53	41	83	128	58	111	61	49	588	46	26
Total size of sequences (bp)	384,714,333	381,568,527	383,971,120	382,043,555	388,473,696	397,125,412	407,596,313	400,990,151	394,174,378	491,646,357	391,869,245	384,741,818
Longest sequence (bp)	25,650,153	33,513,758	31,738,027	29,525,146	22,593,598	39,183,656	39,451,588	32,080,718	44,395,019	44,746,243	44,857,821	43,642,146
Shortest sequence (bp)	31,247	28,273	33,223	25,840	28,179	62,177	60,364	62,282	4,693	31,057	5,656	42,889
Number of sequences > 500 nt (bp)	136 (384,714,333)	53 (381,568,527)	41 (383,971,120)	83 (382,043,555)	128 (388,473,696)	58 (397,125,412)	111 (407,596,313)	61 (400,990,151)	49 (394,174,378)	588 (491,646,357)	46 (391,869,245)	26 (384,741,818)
Number of sequences > 1k nt (bp)	136 (384,714,333)	53 (381,568,527)	41 (383,971,120)	83 (382,043,555)	128 (388,473,696)	58 (397,125,412)	111 (407,596,313)	61 (400,990,151)	49 (394,174,378)	588 (491,646,357)	46 (391,869,245)	26 (384,741,818)
Number of sequences > 10k nt (bp)	136 (384,714,333)	53 (381,568,527)	41 (383,971,120)	83 (382,043,555)	128 (388,473,696)	58 (397,125,412)	111 (407,596,313)	61 (400,990,151)	45 (394,147,392)	588 (491,646,357)	45 (391,863,589)	26 (384,741,818)
Number of sequences > 100k nt (bp)	81 (381,126,077)	32 (380,484,904)	33 (383,439,140)	49 (379,983,247)	104 (386,879,335)	47 (396,216,074)	86 (405,526,260)	50 (400,163,551)	21 (392,844,006)	395 (475,444,831)	18 (390,417,465)	19 (384,284,582)
Number of sequences > 1M nt (bp)	48 (372,884,715)	27 (379,478,749)	20 (380,433,420)	38 (377,675,338)	74 (371,838,746)	16 (391,175,705)	17 (392,624,808)	20 (394,948,352)	15 (391,772,695)	18 (395,640,191)	15 (389,820,951)	16 (383,720,536)
Mean sequence length (bp)	2,828,781	7,199,406	9,365,149	4,602,934	3,034,950	6,846,989	3,672,038	6,573,609	8,044,375	836,133	8,518,896	14,797,762
Median sequence length (bp)	1,399,195	1,015,454	820,882	176,119	1,470,635	141,708	144,367	162,420	75,063	122,237	71,437	15,658,731
N50 sequence length (bp)	11,025,322	22,940,949	22,679,302	17,921,520	7,352,909	26,007,178	27,206,337	21,823,919	29,604,901	26,804,887	30,747,645	27,825,079
L50 sequence count	13	7	7	9	18	7	7	8	6	8	6	6

Supplementary Table 4.

Variety Name/Length(Mb)	Chr01	Chr02	Chr03	Chr04	Chr05	Chr06	Chr07	Chr08	Chr09	Chr10	Chr11	Chr12
CHAO MEO::IRGC 80273-1	44.31	36.54	37.87	35.48	30.35	30.91	29.25	28.52	23.06	23.74	28.80	28.02
Azucena	44.01	36.47	38.43	34.19	30.95	31.19	29.33	29.17	23.13	25.10	30.18	27.49
KETAN NANGKA::IRGC 19961-2	43.44	36.37	37.49	34.28	30.41	31.74	30.62	29.55	23.54	25.90	30.70	26.71
ARC 10497::IRGC 12485-1	43.68	35.92	38.38	34.33	30.57	31.84	29.53	29.51	24.22	24.63	29.24	26.63
IR 64	44.35	37.11	38.83	35.28	31.01	32.15	31.20	30.18	24.27	25.56	31.22	25.54
PR 106::IRGC 53418-1	44.39	38.15	39.18	36.99	30.84	32.21	30.66	30.03	24.98	25.71	32.03	26.01
LIMA::IRGC 81487-1	44.96	37.90	39.45	36.94	31.25	32.48	30.74	29.29	24.05	25.77	32.42	27.37
KHAO YAI GUANG::IRGC 65972-1	44.76	37.53	40.37	36.89	31.27	32.08	30.27	29.25	25.34	25.44	32.70	27.83
GOBOL SAIL (BALAM)::IRGC 26624-2	44.40	37.06	39.50	37.68	30.84	32.49	30.32	29.60	24.95	25.67	31.75	27.50
LIU XU::IRGC 109232-1	44.75	37.64	39.28	37.89	30.94	32.35	29.70	30.91	24.01	25.46	32.30	26.80
LARHA MUGAD::IRGC 52339-1	44.86	36.83	39.14	36.68	30.75	32.76	30.44	29.62	24.43	25.26	32.11	27.34
NATEL BORO::IRGC 34749-1	43.64	37.78	38.83	35.33	31.01	31.93	29.23	29.95	23.59	25.10	31.31	26.02

Supplementary Table 5.

Variety Name	CHAO MEO::IRGC 80273-1	Azucena	KETAN NANGKA::I RGC 19961-	ARC 10497::IRGC 12485-1	IR 64	PR 106::IRGC 53418-1	LIMA::IRGC 81487-1	KHAO YAI GUANG::IR GC 65972-1	GOBOL SAIL (BALAM)::I	LIU XU::IRGC 109232-1	LARHA MUGAD::IR GC 52339-1	NATEL BORO::IRG C 34749-1
Count	24	17	22	22	18	22	22	27	24	56	37	18
Min length (Mbp)	0.41	0.62	0.39	0.29	0.56	0.63	0.58	0.46	0.47	0.51	0.75	0.61
Median length (Mbp)	17.35	23.91	20.01	19.26	24.25	18.26	19.38	13.87	17.99	3.36	3.15	22.37
Mean length (Mbp)	15.98	22.65	17.93	17.51	21.93	18.25	18.55	15.38	16.65	9.01	11.67	21.55
N50 length (Mbp)	22.75	26.69	26.75	25.29	26.66	27.06	26.45	31.45	25.99	22.30	27.09	25.96
Max length (Mbp)	35.93	38.49	34.48	32.16	39.40	39.28	37.32	40.84	37.90	31.18	36.59	35.77
Total length (Mbp)	383.45	385.08	394.42	385.13	394.76	401.43	408.06	415.35	399.52	504.52	431.94	387.98
Mapping Ratio (Contigs/Maps)	100.00	99.09	97.30	99.24	98.14	98.62	100.00	96.46	98.51	97.59	90.40	98.31

Supplementary Table 6.

Variety Name (subpopulation)	SV	Nipponbare (GJ-temp)	CHAO MEO::IRGC 80273-1 (GJ-subtrp)	Azucena (GJ-trop1)	KETAN NANGKA::IRGC 19961-2 (GJ-trop2)	ARC 10497::IRGC 12485-1 (cB)	PR 106::IRGC 53418-1 (XI-1B2)	Minghui 63 (XI-adm)	IR 64 (XI-1B1)	Zhenshan 97 (XI-1A)	LIMA::IRGC 81487-1 (XI-3A)	KHAO YAI GUANG::IRGC 65972-1 (XI-3B1)	GOBOL SAIL (BALAM)::IRGC 26624-2 (XI-2A)	LIU XU::IRGC 109232-1 (XI-3B2)	LARHA MUGAD::IRGC 52339-1 (XI-2B)	N 22::IRGC 19379-1 (cA1)	NATEL BORO::IRGC 34749-1 (cA2)
Nipponbare	Number	-															
(GJ-temp)	Size/Mb	-															
CHAO MEO::IRGC 80273-1	Number	13,530	-														
(GJ-subtrp)	Size/Mb	19.06	-														
Azucena	Number	15,227	12,626	-													
(GJ-trop1)	Size/Mb	21.27	17.94	-													
KETAN NANGKA::IRGC 19961-2	Number	16,516	15,381	13,401	-												
(GJ-trop2)	Size/Mb	23.07	21.66	18.55	-												
ARC 10497::IRGC 12485-1	Number	20,532	19,224	19,337	19,654	-											
(cB)	Size/Mb	26.82	25.65	26.78	27.16	-											
PR 106::IRGC 53418-1	Number	29,490	28,286	28,018	27,680	27,051	-										
(XI-1B2)	Size/Mb	39.16	38.61	38.39	39.54	36.61	-										
Minghui 63	Number	29,560	28,664	27,993	27,866	27,961	12,567	-									
(XI-adm)	Size/Mb	39.21	38.38	38.38	38.88	39.33	19.62	-									
IR 64	Number	30,236	29,042	28,568	28,132	27,577	11,346	14,231	-								
(XI-1B1)	Size/Mb	39.64	39.06	39.26	39.66	38.31	17.57	23.72	-								
Zhenshan 97	Number	29,338	28,478	28,070	27,705	26,985	14,686	16,467	15,023	-							
(XI-1A)	Size/Mb	39.96	39.81	40.71	40.27	38.46	23.99	26.02	23.77	-							
LIMA::IRGC 81487-1	Number	30,606	29,594	29,341	28,411	28,083	16,882	15,598	16,440	17,576	-						
(XI-3A)	Size/Mb	40.32	39.79	40.43	40.57	39.51	26.69	25.75	24.89	28.76	-						
KHAO YAI GUANG::IRGC 65972-1	Number	31,160	30,026	29,549	29,344	28,568	15,697	14,477	16,398	16,532	14,467	-					
(XI-3B1)	Size/Mb	41.54	40.63	41.37	42.15	40.66	25.30	22.18	26.86	28.33	23.41	-					
GOBOL SAIL (BALAM)::IRGC 26624-2	Number	30,865	29,947	29,403	29,140	27,857	16,000	15,292	15,684	16,778	14,693	14,398	-				
(XI-2A)	Size/Mb	41.02	40.75	40.56	41.51	39.06	25.22	24.40	25.45	27.91	23.57	23.50	-				
LIU XU::IRGC 109232-1	Number	32,002	31,022	30,806	30,416	29,422	17,928	18,033	18,149	17,381	18,113	17,793	18,041	-			
(XI-3B2)	Size/Mb	41.03	40.34	40.96	40.79	39.24	25.83	26.19	25.62	25.94	25.75	26.38	26.79	-			
LARHA MUGAD::IRGC 52339-1	Number	33,364	32,549	31,962	31,695	30,746	19,682	20,556	20,489	20,070	20,663	20,141	19,477	21,956	-		
(XI-2B)	Size/Mb	41.19	39.99	39.95	40.65	38.17	26.59	28.49	28.19	28.84	28.55	28.94	26.56	28.14	-		
N 22::IRGC 19379-1	Number	29,317	28,677	27,851	28,291	26,367	23,365	23,987	23,423	23,480	24,152	24,087	22,967	25,437	25,239	-	
(cA1)	Size/Mb	39.54	39.25	39.04	40.09	35.67	35.80	37.42	36.17	37.32	37.81	38.85	36.50	38.32	35.76	-	
NATEL BORO::IRGC 34749-1	Number	34,908	34,302	34,004	34,295	32,015	29,623	29,401	29,209	29,944	29,809	29,528	28,308	31,302	30,840	20,265	-
(cA2)	Size/Mb	39.54	39.73	39.24	39.89	36.59	38.90	38.33	39.64	40.63	40.23	40.51	37.94	40.10	36.50	23.88	-

Subpopulations: GJ = *Geng*-japonica where temp = temperate, subtrp = subtropical, trop = tropical; cB = *circum*-Basmati; XI = *Xian*-indica; adm = admixed; cA = *circum*-Aus

Supplementary Figure 1

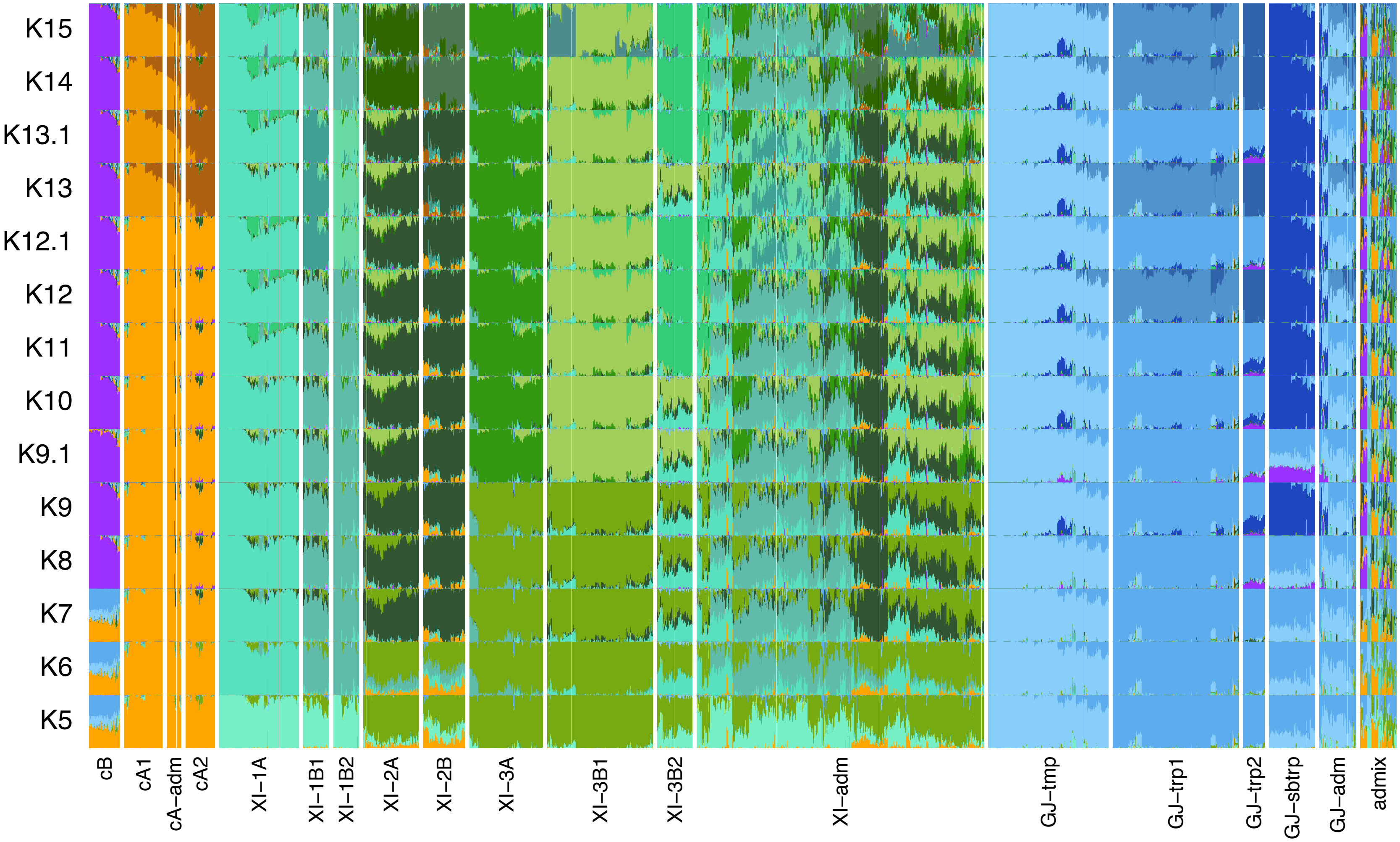

Supplementary Figure 2

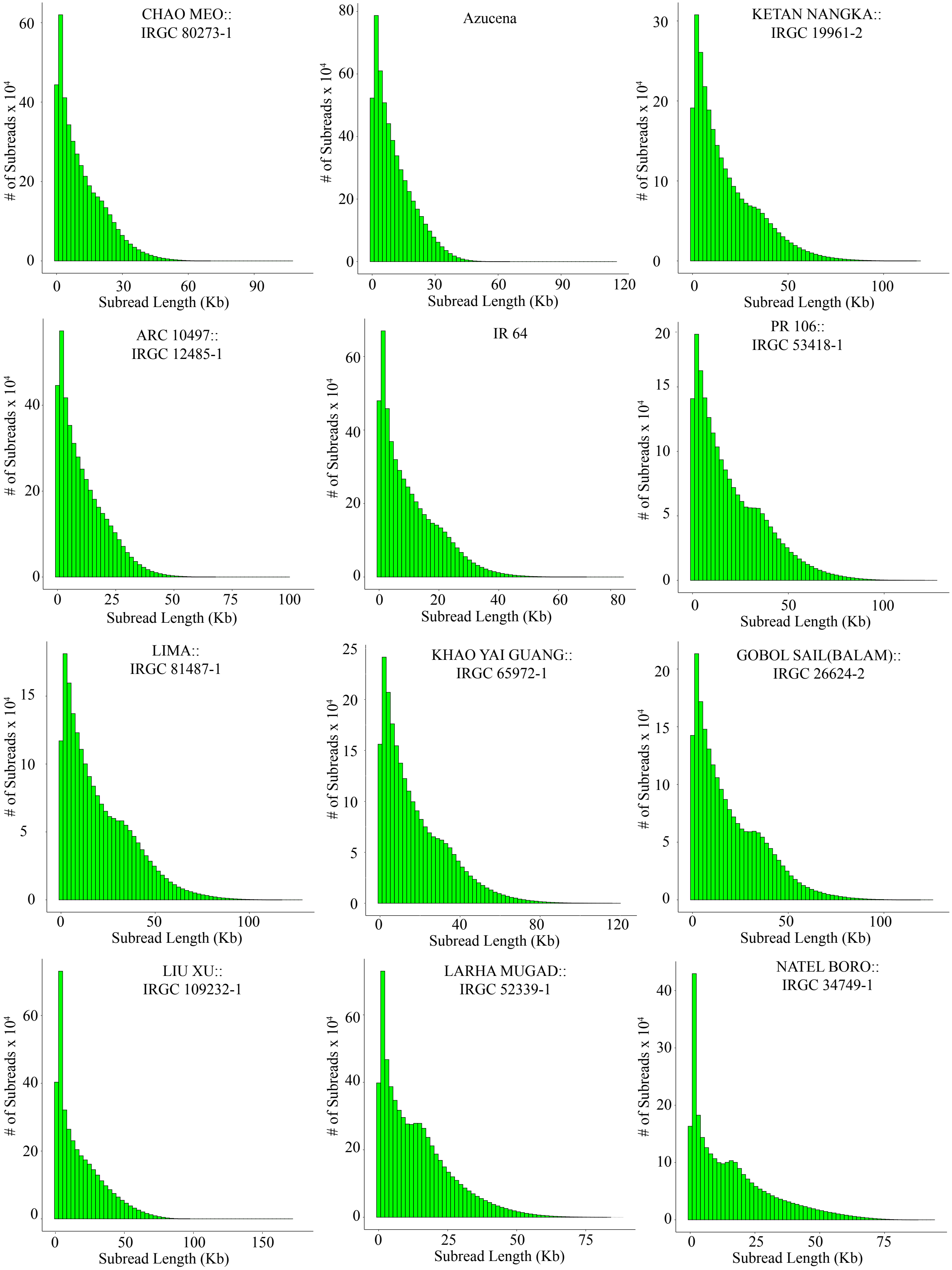

Supplementary Figure 3

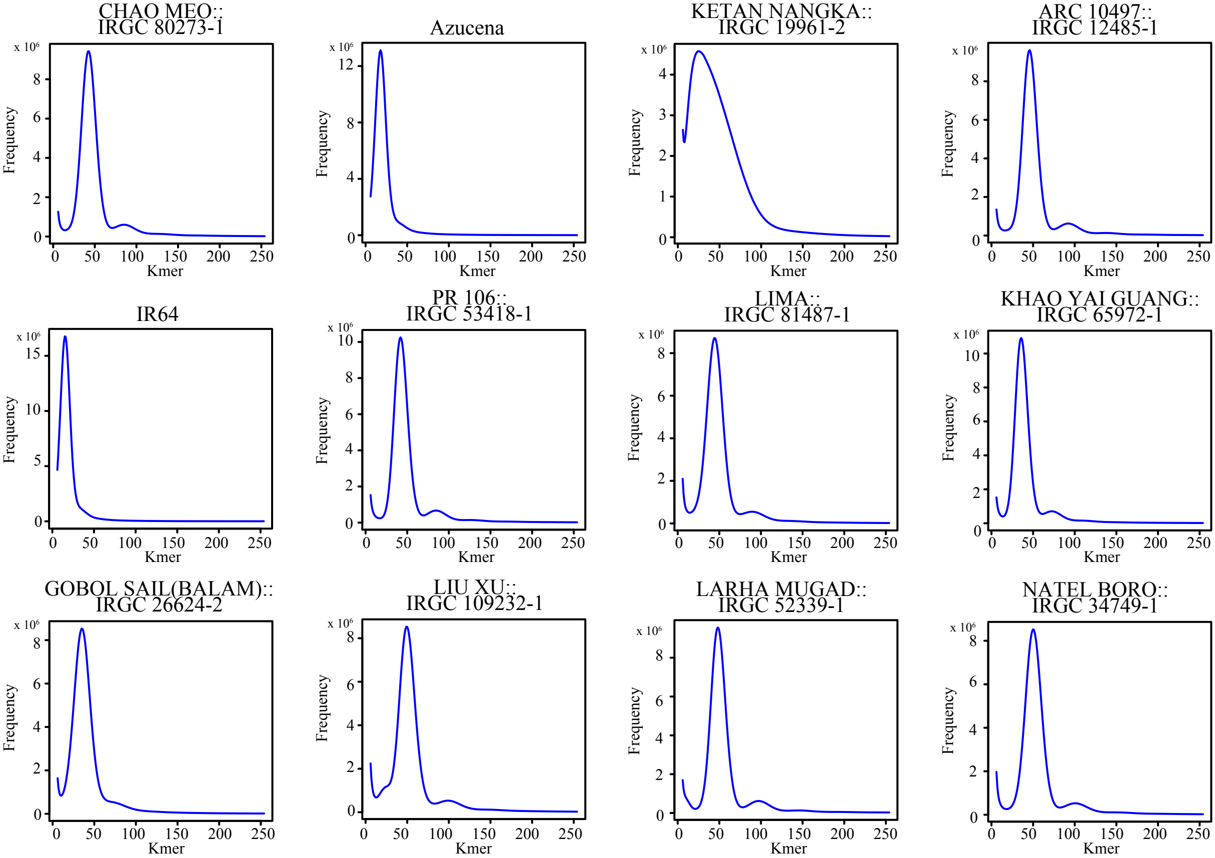

Supplementary Figure 4a. CHAO MEO::IRGC 80273-1

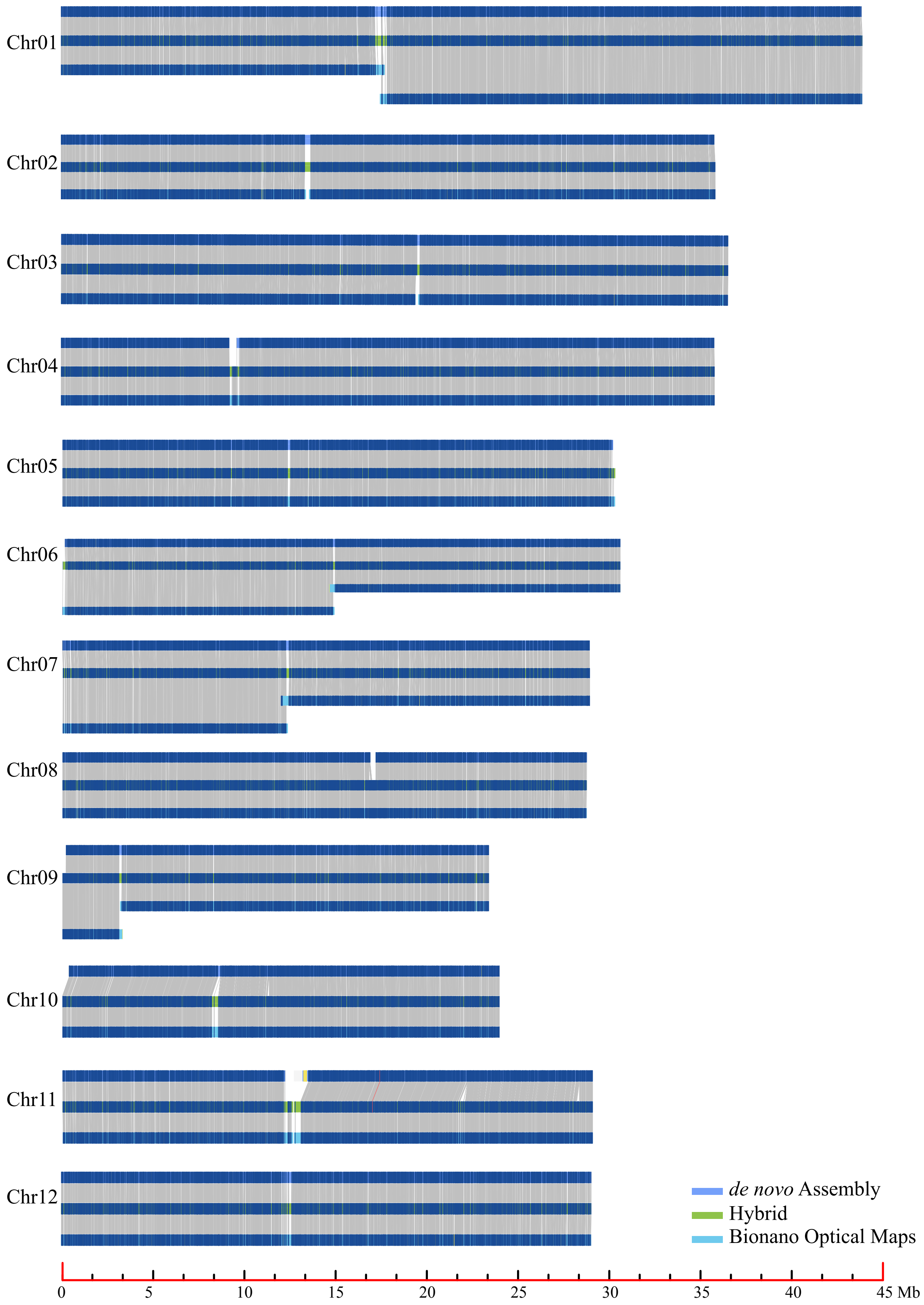

Supplementary Figure 4b. Azucena

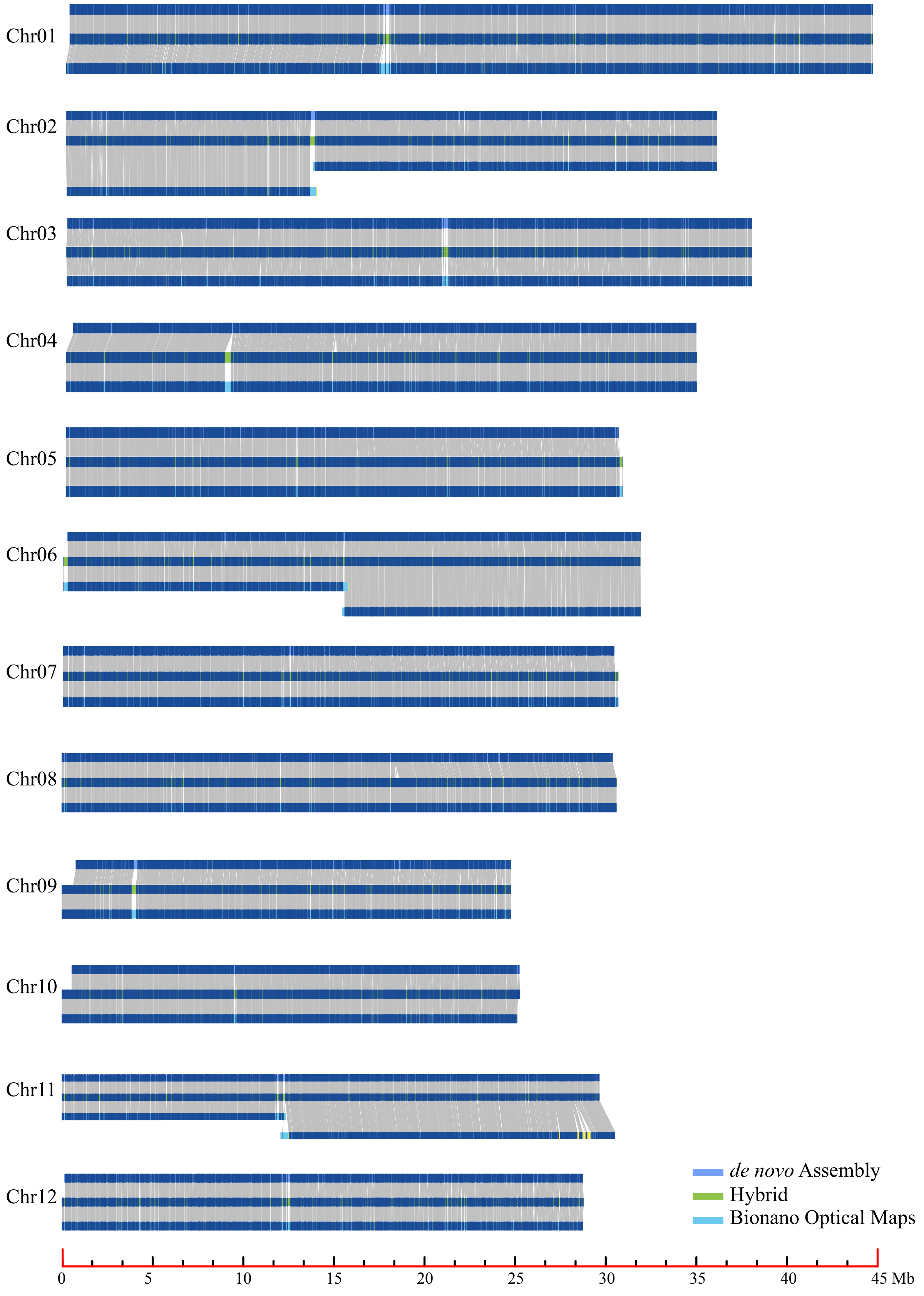

Supplementary Figure 4c. KETAN NANGKA::IRGC 19961-2

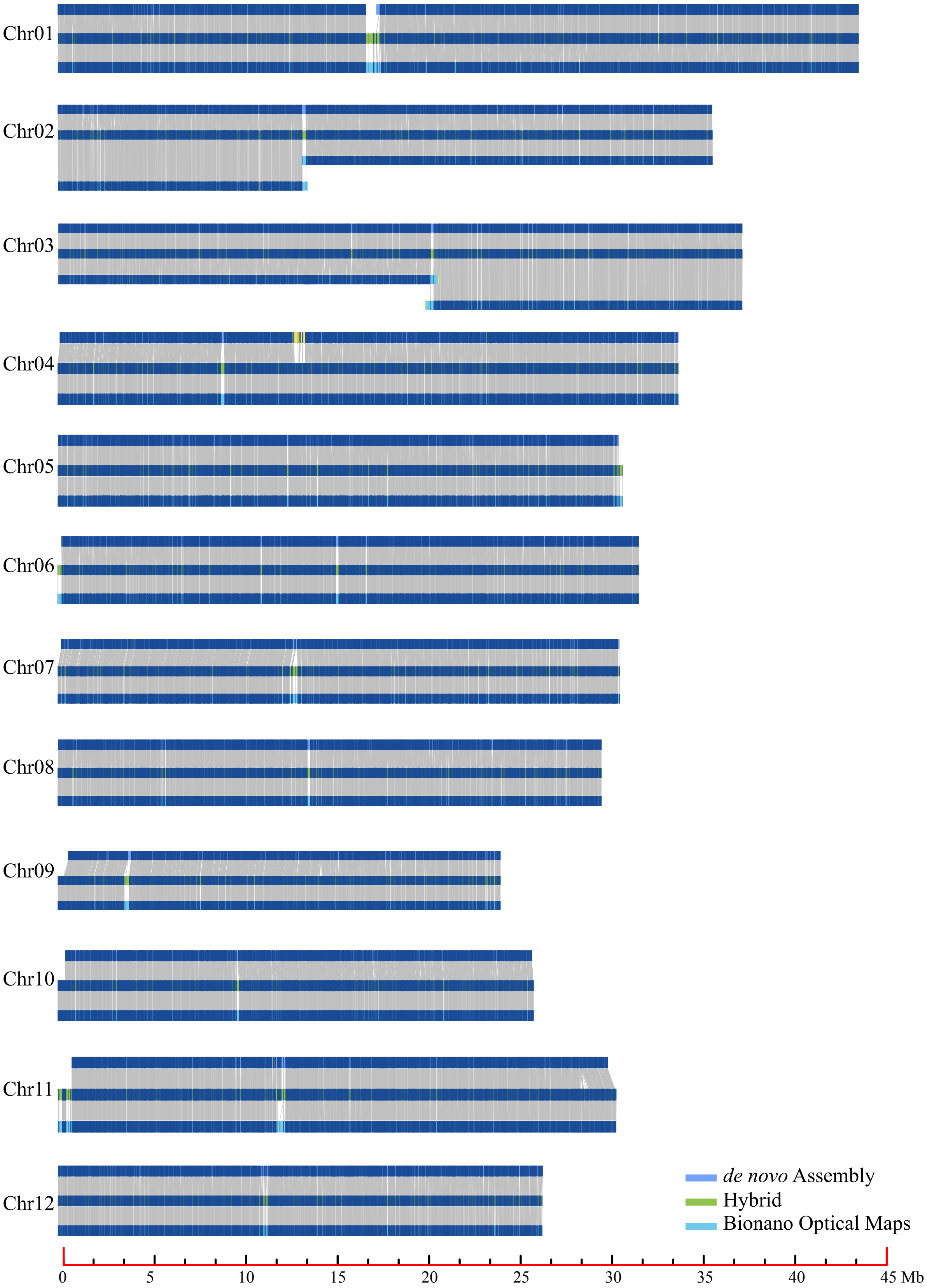

Supplementary Figure 4d. ARC 10497::IRGC 12485-1

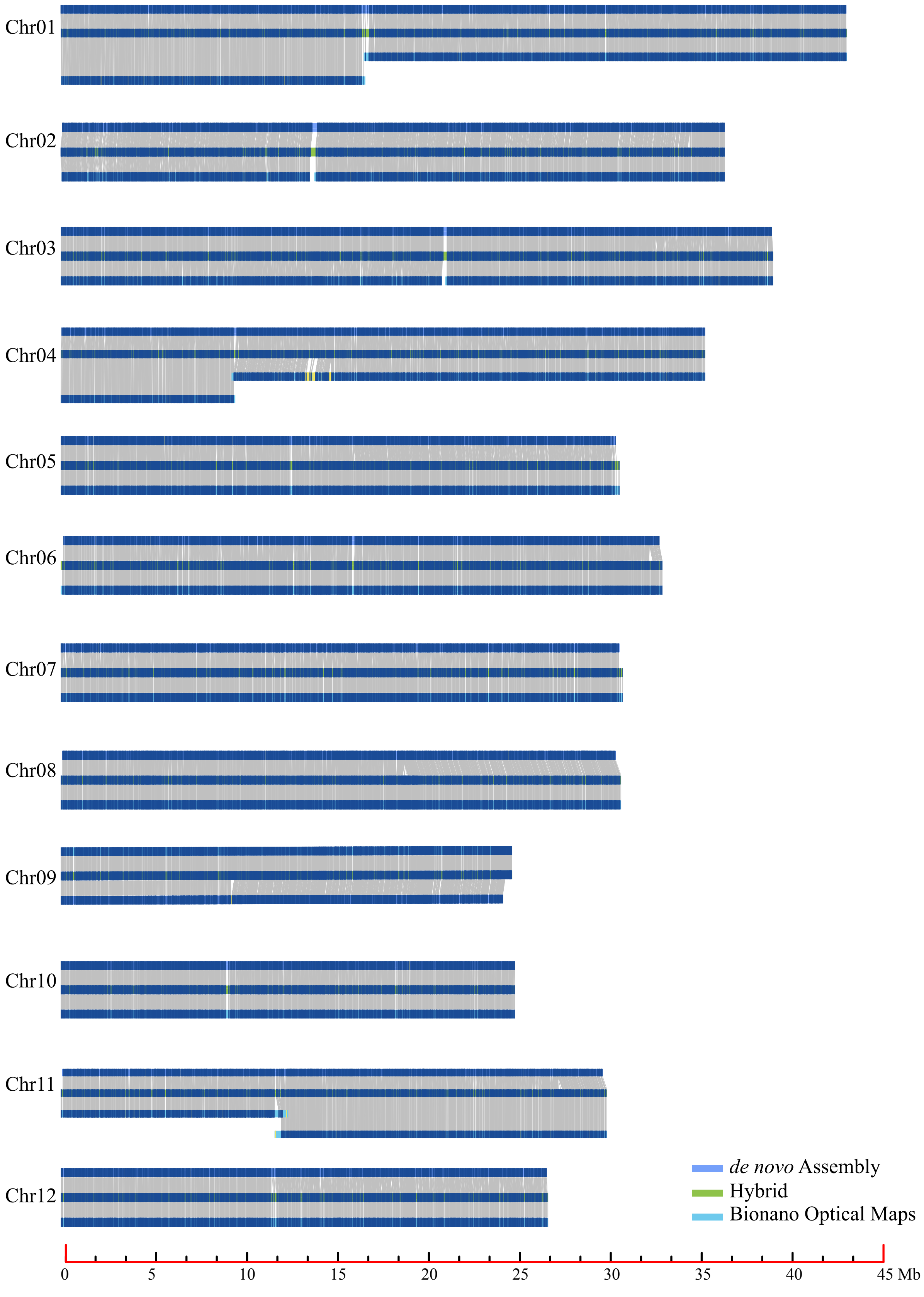

Supplementary Figure 4e. IR64

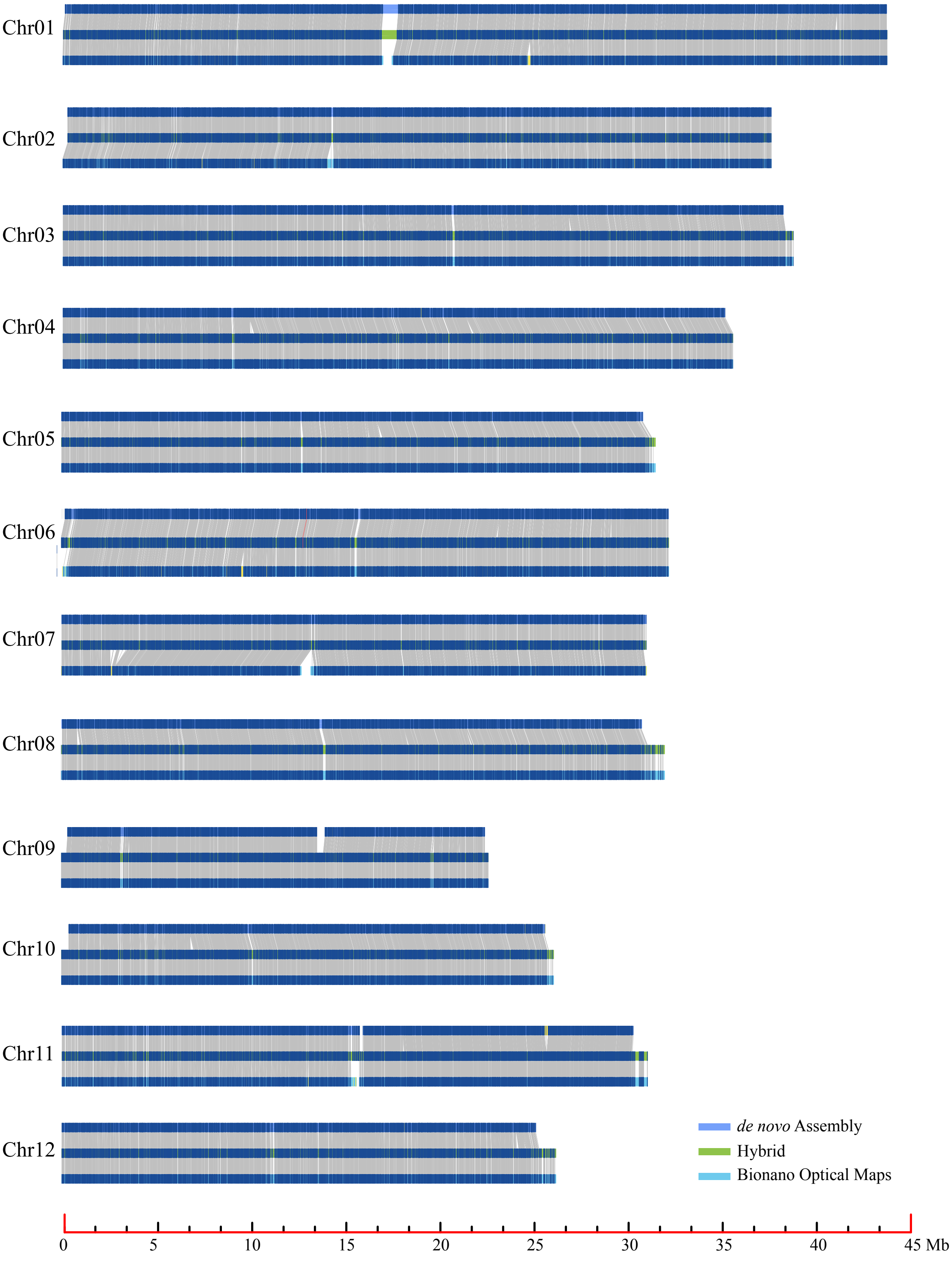

Supplementary Figure 4f. PR 106::IRGC 53418-1

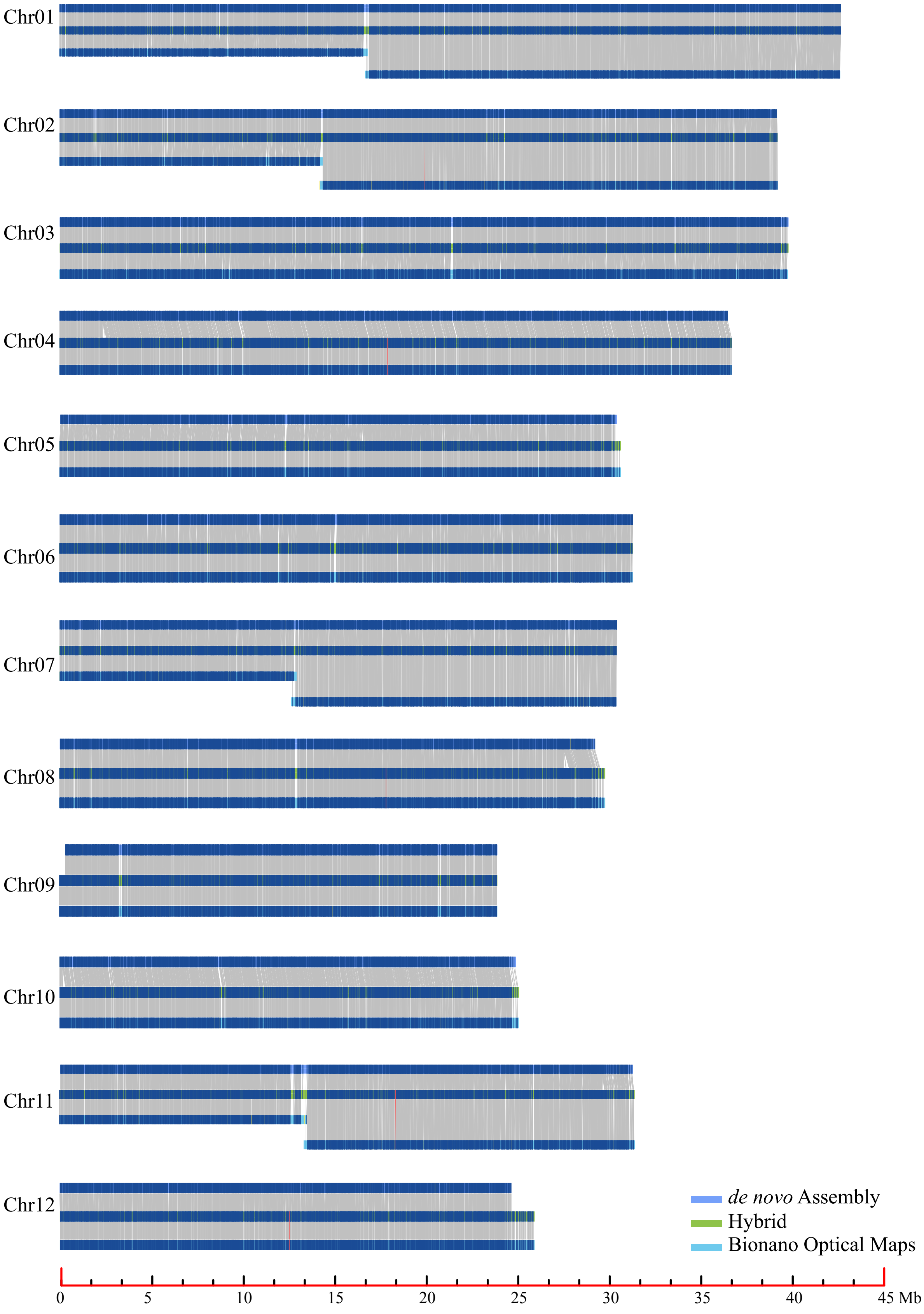

Supplementary Figure 4g. LIMA::IRGC 81487-1

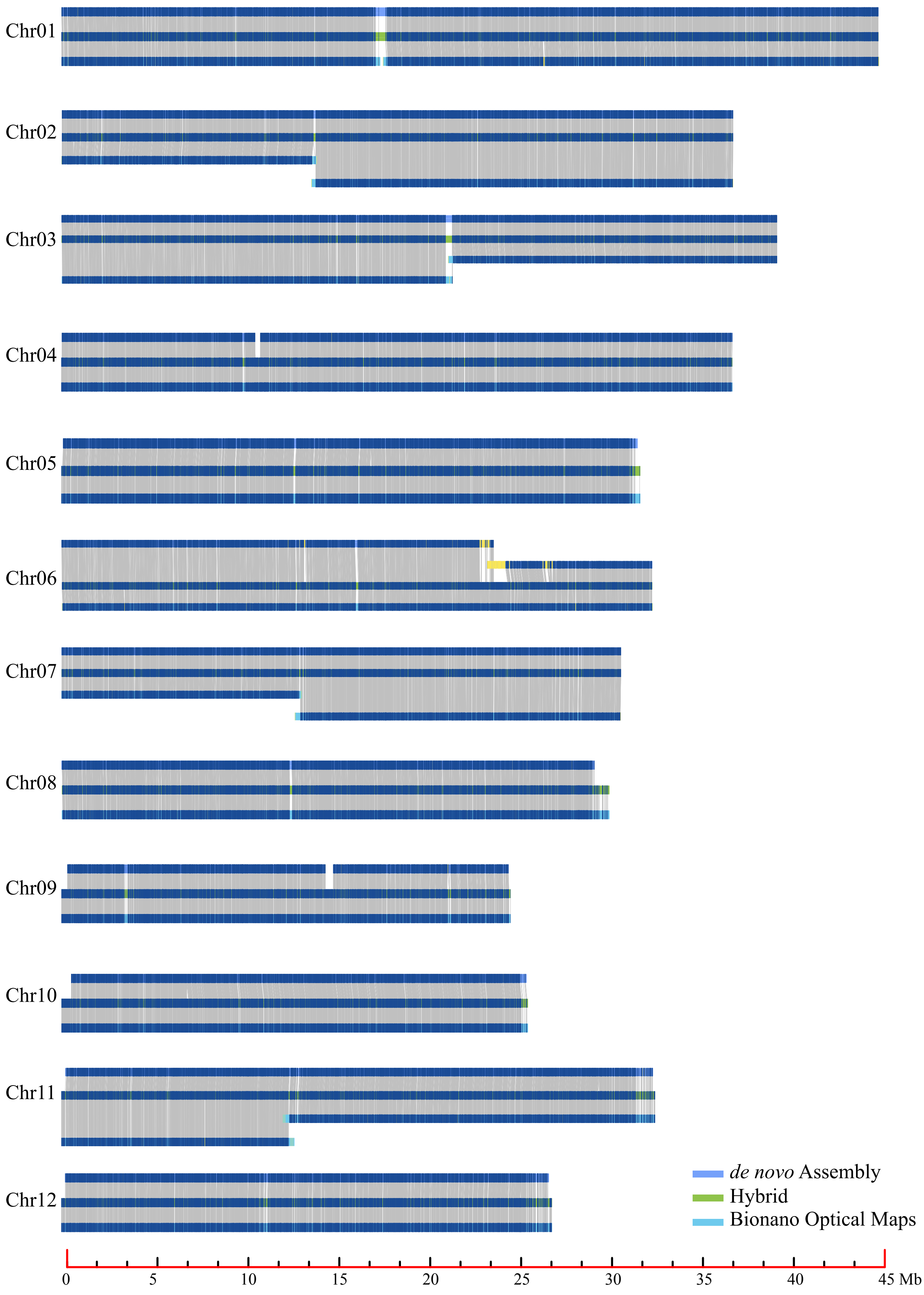

Supplementary Figure 4h. KHAO YAI GUANG::IRGC 65972-1

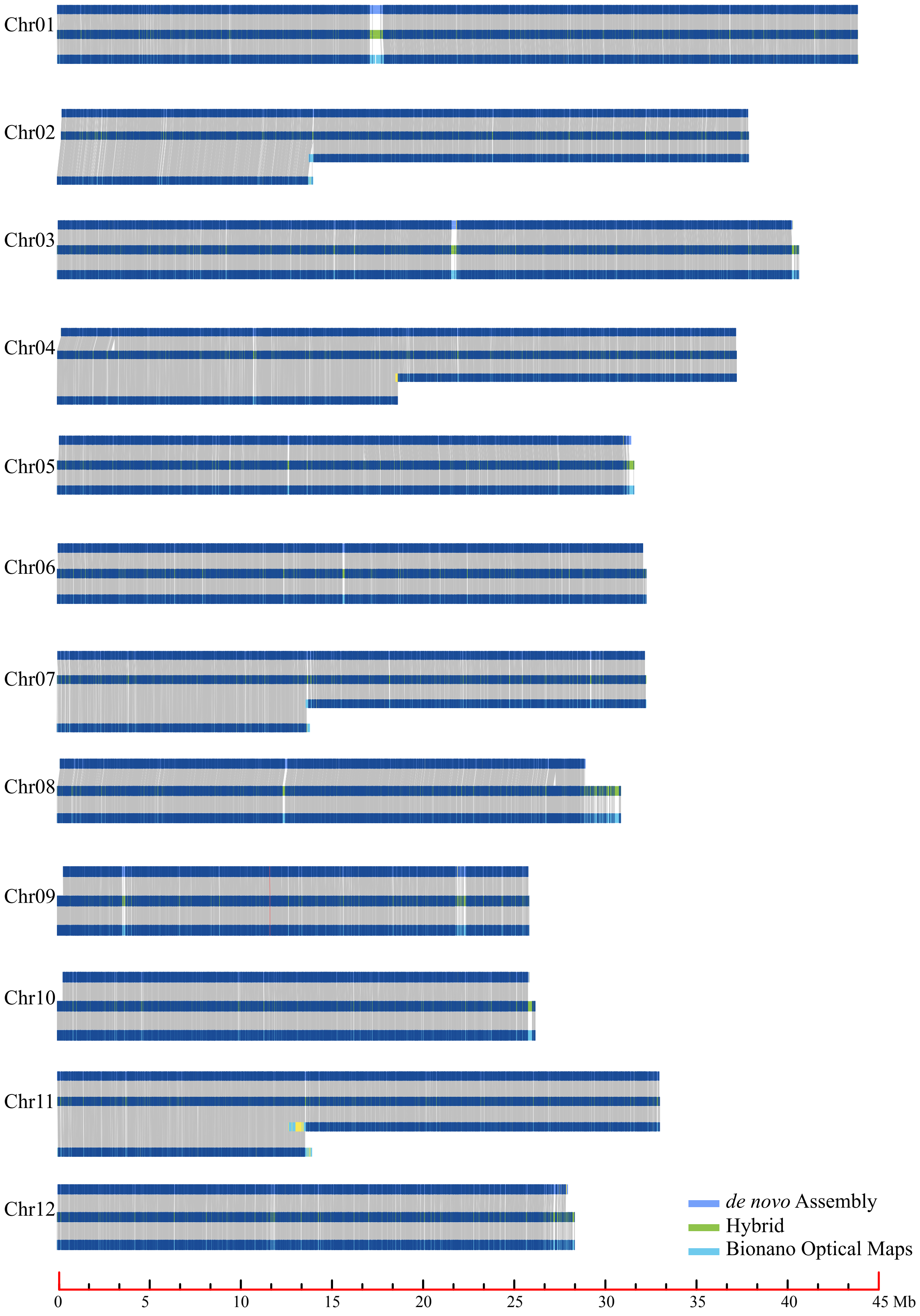

Supplementary Figure 4i. GOBOL SAIL (BALAM)::IRGC 26624-2

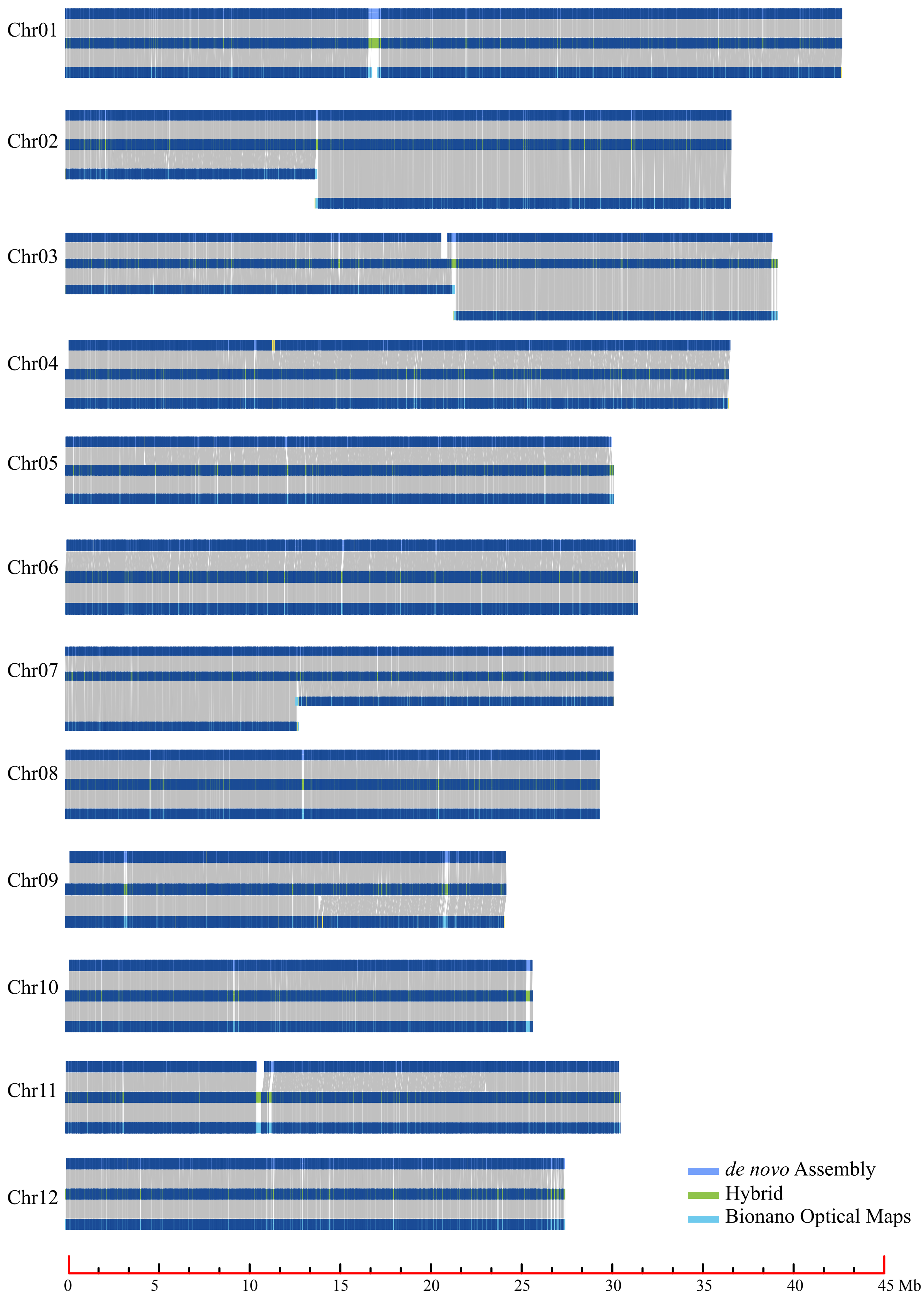

Supplementary Figure 4j. LIU XU::IRGC 109232-1

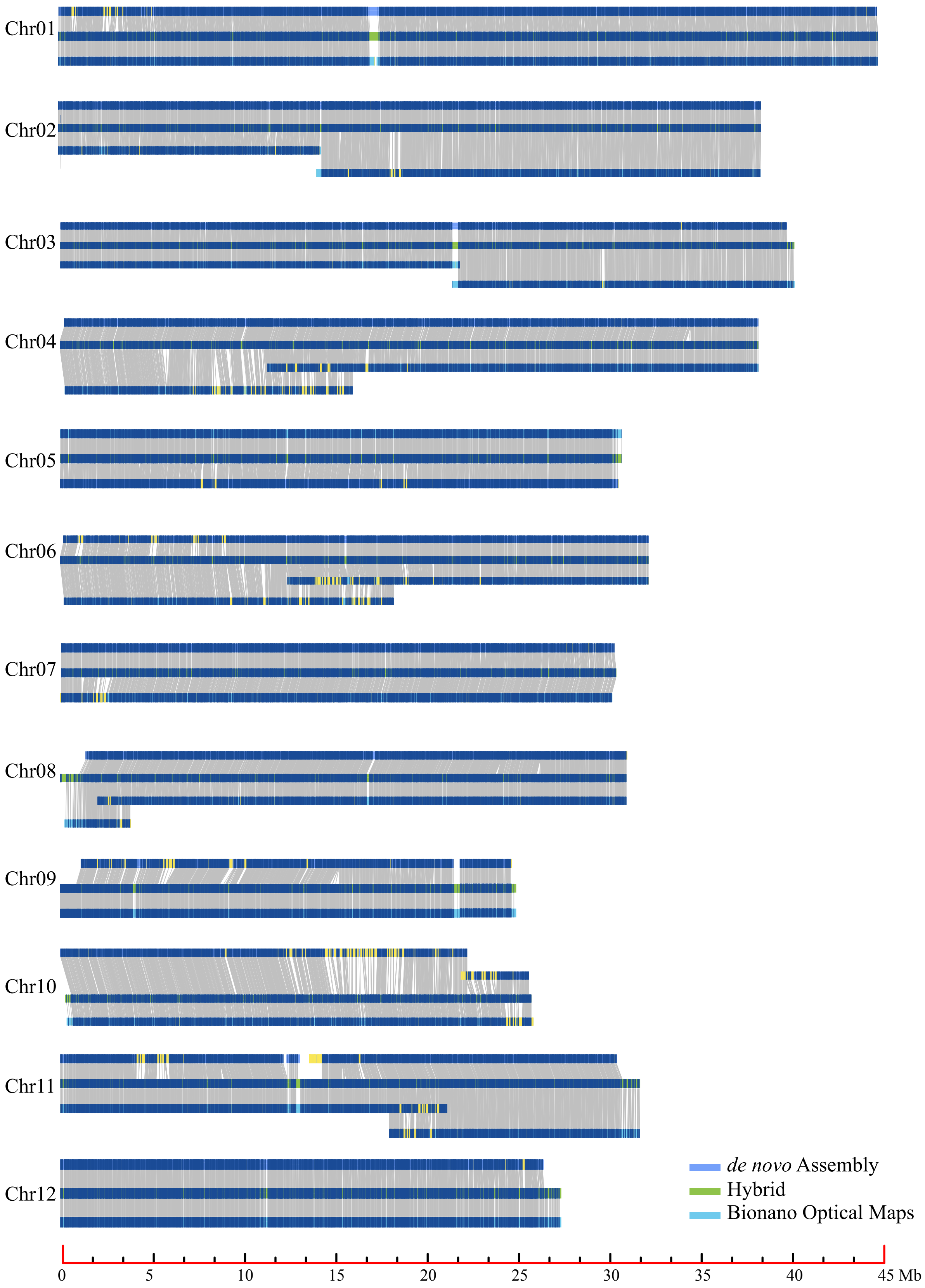

Supplementary Figure 4k. LARHA MUGAD::IRGC 52339-1

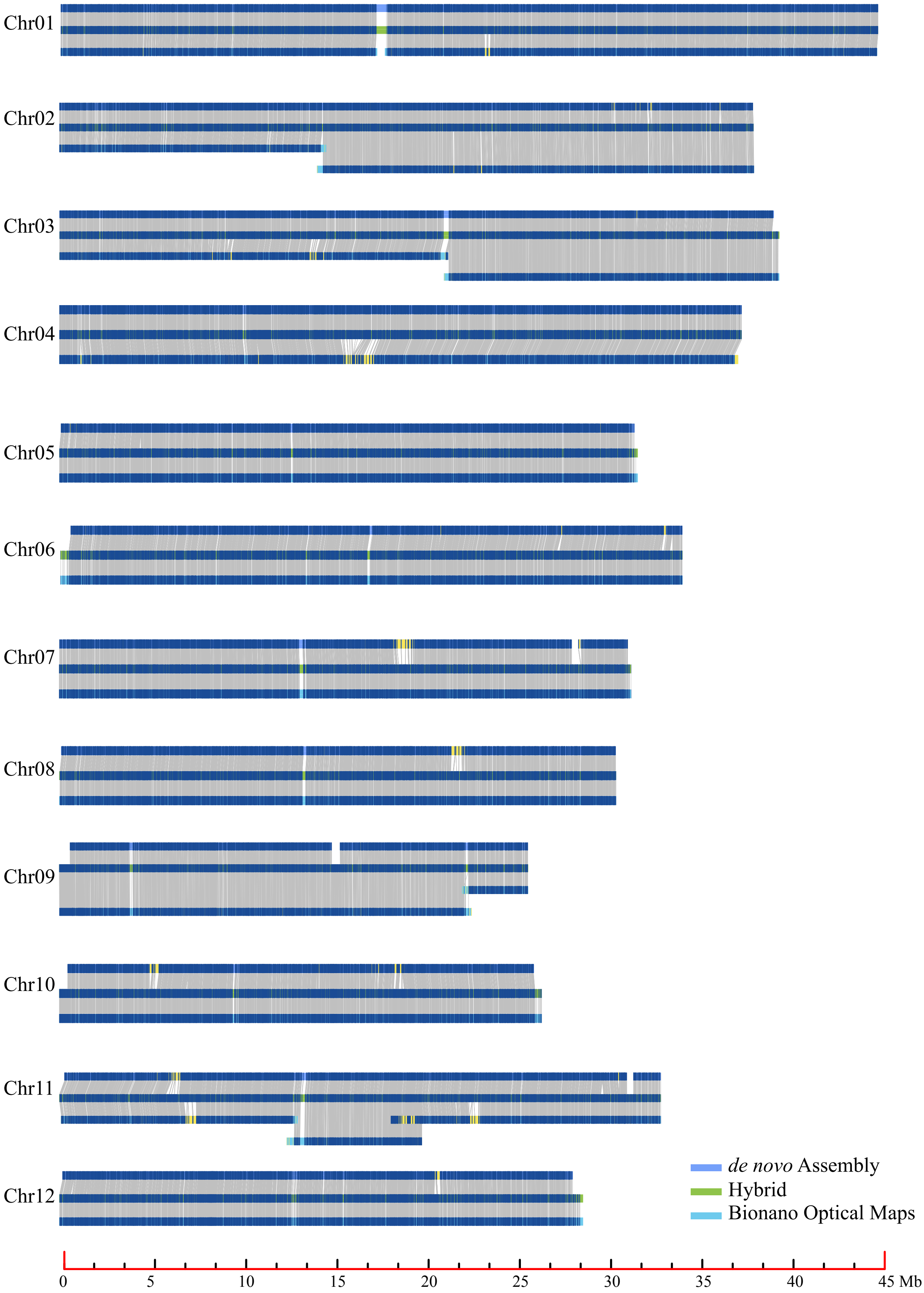

Supplementary Figure 4l. NATEL BORO::IRGC 34749-1

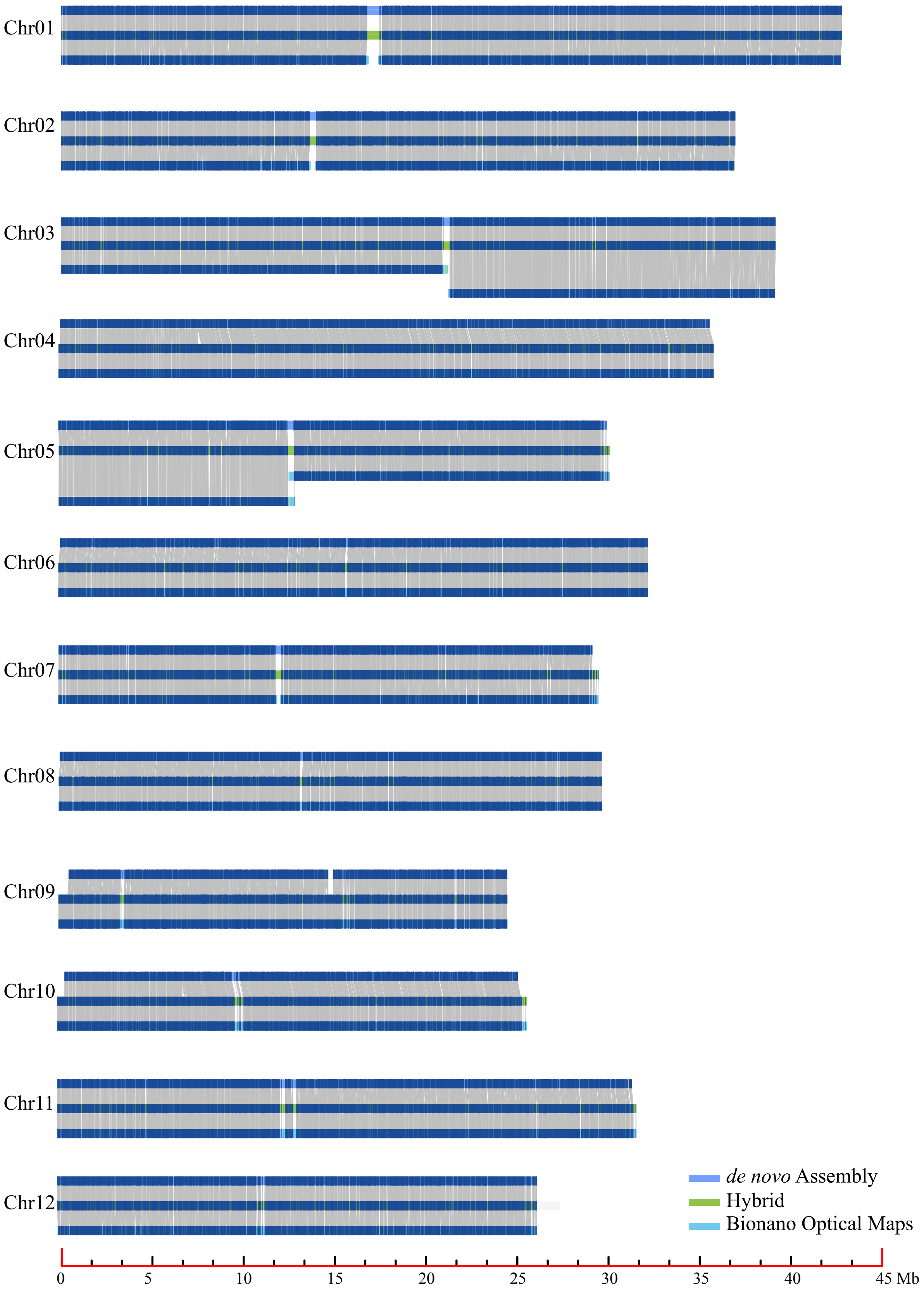

Supplementary Figure 5

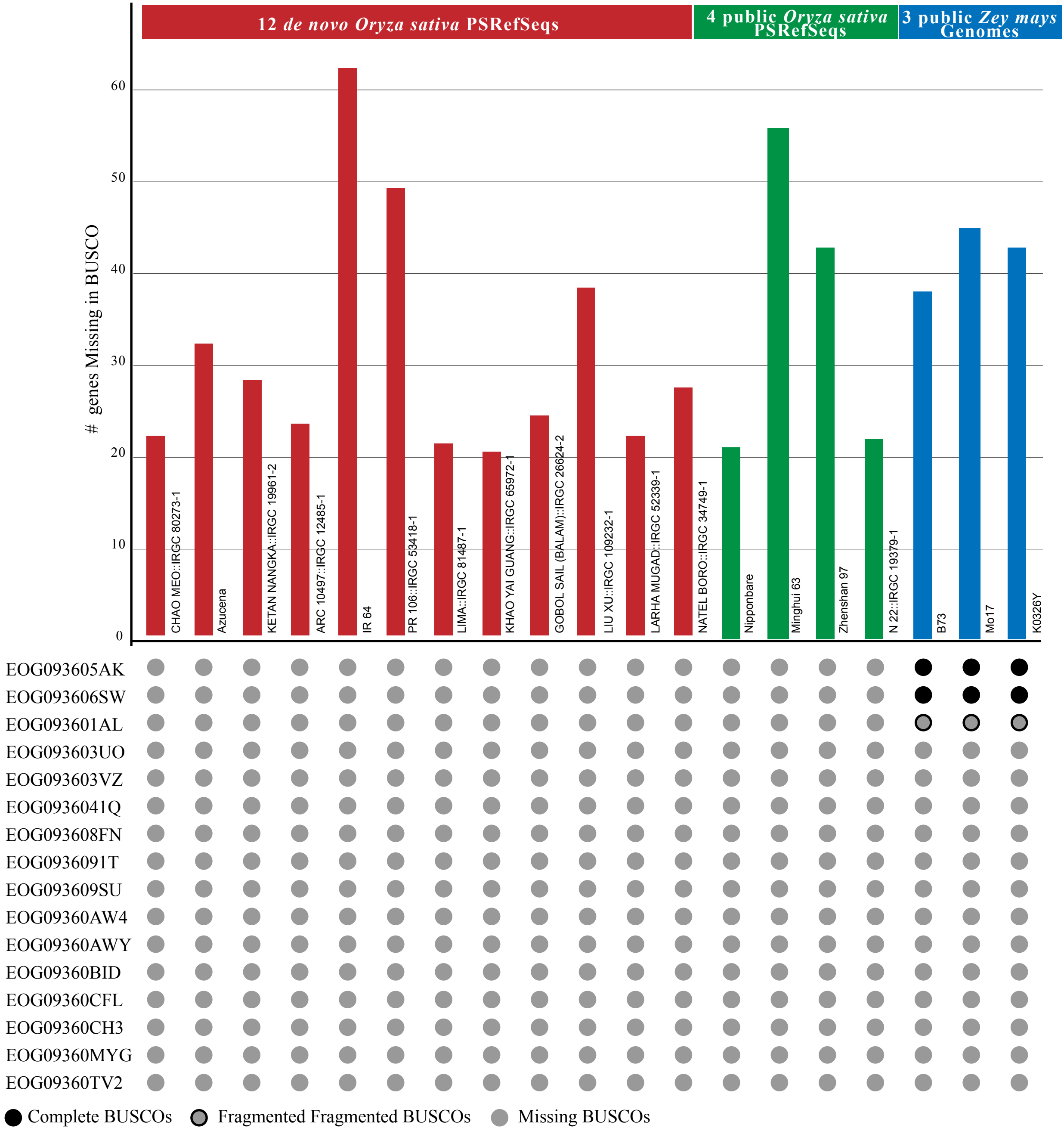

Supplement: Supplementary file 1 — Supplementary information [file 41597_2020_438_MOESM1_ESM.pdf]
